# Recyclable Robust Plastic Scintillation Resin Achieving the Exceptional Separation and Detection of Technetium‐99

**DOI:** 10.1002/advs.202411523

**Published:** 2024-11-05

**Authors:** Tonghuan Liu, Yichen Hu, Junqiang Yang, Kesheng Hu, Bei Qi, Yun Zhou, Juan Tong, Man Wang, Liang Huang, Keliang Shi, Xiaolin Hou

**Affiliations:** ^1^ Frontier Science Center for Rare Isotopes, School of Nuclear Science and Technology Lanzhou University Lanzhou 730000 China; ^2^ Wuhan National Laboratory for Optoelectronics Huazhong University of Science and Technology Wuhan 430074 China

**Keywords:** adsorption, detection, mechanism, plastic scintillation resin, technetium/rhenium

## Abstract

Rapid detection and absorption of ^99^TcO_4_^⁻^ contamination in the environment are critical due to its high radioactivity, long half‐life, and significant environmental mobility. Resins have been demonstrated effective bifunctional properties for both the detection and separation of ^99^TcO_4_^⁻^. However, the poor stability of these compounds limits their practical application. Here, a chemical grafting strategy is presented to synthesize ultra‐stable plastic scintillation resin, in which 4‐vinylpyridine and divinylbenzene are cross‐linked as matrix polymer to withstand extreme conditions and a fluorophore “shield” to convert beta radiation into detectable signals. As expected, the as‐obtained resin exhibits a high adsorption capacity of 549.2 mg g^⁻¹^ for ^99^TcO_4_^⁻^ with a rapid kinetic response of just 10 min as well as superior selectivity at 1000 times excess of interfering ions and full reusability. Moreover, it showed remarkable stability under 800 kGy, 3.0 mol L^−1^ HNO_3_ or 2.5 L solution continuous leaching, consistently maintaining high separation and detection efficiency after recycling 10 times. This strategy paves a new way to develop stable resin for the rapid capture and accurate measurement of ^99^TcO_4_^⁻^, which owns great potential for practical application.

## Graphical abstract

In this work, a class of super‐stable soldier‐like scintillation resin is prepared by quaternization reaction, and C2 PSresin with comprehensive properties is selected by experiment and theoretical calculation, which can be used in the rapid capture and analysis of ^99^Tc samples.

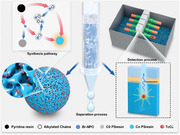

## 1 Introduction

Technetium‐99 (^99^Tc) is a pure β‐emitting radionuclide with a relatively long half‐life (t_1/2_ = 2.1 × 10^5^a), primarily produced through nuclear fission of ^235^U and ^239^Pu at a high fission yield of 6.06%.^[^ ^1^ ^]^ It occupies a pivotal position in the realms of spent fuel reprocessing, radioactive waste disposal, and environmental radioactivity monitoring. Notably, the release of ^99^Tc predominantly emanates from nuclear reprocessing facilities, nuclear weapons tests, and nuclear accidents.^[^ ^2^ ^]^ For instance, ≈700 Ci of Tc has been released into the subsurface in the Hanford Site (Washington State, U.S.A.).^[^ ^3^ ^]^ Due to its high solubility and strong environmental migration capabilities, TcO_4_^−^ can pose potential hazards and threats to the natural environment. Hence, rapid analysis of released ^99^Tc into the environment is crucial to promptly manage contamination levels and mitigate further dissemination. ^[^ ^4^ ^]^ View from this insight, establishing a tailored emergency analysis framework for nuclear incidents is imperative.

Currently, many methodologies have been established for the analysis of ^99^Tc samples, spanning several techniques such as spectrophotometry and inductively coupled plasma optical emission spectrometer (ICP‐OES)^[^ ^5^ ^]^ based on spectral analysis, various mass spectrometry techniques including mass analysis such as accelerator mass spectrometry (AMS),^[^ ^6^ ^]^ resonance ionization mass spectrometry (RIMS)^[^ ^7^ ^]^ and inductively coupled plasma mass spectrometry (ICP‐MS),^[^ ^8^ ^]^ as well as Geiger‐Miller (GM) counter^[^ ^9^ ^]^ and liquid scintillation counter (LSC)^[^ ^10^ ^]^ based on radiation measurement. However, these methods usually require robust radiochemical separations of ^99^Tc to effectively mitigate the influence of matrix elements, isobaric interferences, and interfering radionuclides present within environmental matrices.^[^ ^11^ ^]^

Typically, the separation and detection steps are independent of each other in the traditional analytical procedures. The cumbersome operation step not only prolongs the analysis time, poor selectivity, high cost, and hazardous wastes were produced, but also introduces unpredictable errors. Consequently, these procedures fall short of meeting the stringent requirements for both time efficiency and accuracy crucial for emergency measurements.^[^ ^12^ ^]^

In order to overcome the issue in the disposal of additional waste generated in the separation and analysis of nuclides TcO_4_^−^ through the one‐pot method, it is better to use plastic scintillation resin (PSresin), which permits unification of the extraction chromatography with extraction group and scintillation with detection measurement steps. In practical application, the extraction group selectively captures radionuclides in aqueous solutions, channeling the subsequent release of radionuclide decay energy into the polymer matrix. This energy transfer excites the scintillator to a higher energy state, which upon de‐excitation, emits fluorescence detected by photomultiplier tubes (PMT), thus achieving adsorption and detection at one time.^[^ ^13^ ^]^ Notably, DeVol et al. synthesized PSresin via impregnation, showcasing high detection efficiency for ^99^Tc initially, however, subsequent loading/elution trials using 8 mol L^−1^ HNO_3_ saw a decline in efficiency from 50% to 1%, which could not be used in a strong nitric acid environment.^[^ ^14^ ^]^ At present, most of the PSresins for detecting ^99^Tc were chosen Aliquat‐336 as the extractant, 2,5‐Diphenyloxazole (PPO) as the fluorophore, and 1,4‐bis‐2(5‐phenyloxazoyl) benzene (POPOP) as the wave shifting agent, which including commercialized PSresin (TK‐TcScint).^[^ ^14^, ^15^ ^]^ The scintillators PPO and POPOP would leach in high concentration HNO_3_ (> 0.1 mol L^−1^), leading to stability deviations, and decreasing the detection efficiency. Additionally, as the selectivity of the extractant is not considered, the measurement of ^99^Tc on TK‐TcScint usually had poor removal ability for interfering nuclides ^36^Cl and ^239^Pu.^[^ ^15^, ^16^ ^]^ and other radionuclides may cause interference.^[^ ^12^ ^]^ Obviously, this way of physical binding may affect the resin recycling efficiency in extreme environments such as strong radioactivity (β, γ and neutron radiation, etc.), high acid content, or high salt content.

Recently, DeVol et al, synthesized PSresin via atom‐transfer radical grafting on a thin polymer layer aimed to address fluor molecule leaching concerns, yet limitations in surface polymerization effect and decreased counting rates still remain.^[^ ^17^ ^]^ The synthesis method is too complex because the extractant and scintillation agent need to be separated by a polymer film, while the thickness and particle size of the polymer film will affect the detection efficiency of the PSresin.^[^ ^18^ ^]^ Therefore, the challenge of developing the PSresin is to improve the selective separation efficiency and the stability of scintillators of the resin toward ^99^Tc, especially in complex environments such as high acidity, high salinity, and strong irradiation conditions, even at continuous flushing of large volume solution, so as to ensure that most of the ^99^Tc in the sample can be retained on the plastic scintillating resin to improving its detection efficiency.^[^ ^19^ ^]^

**Figure 1:**
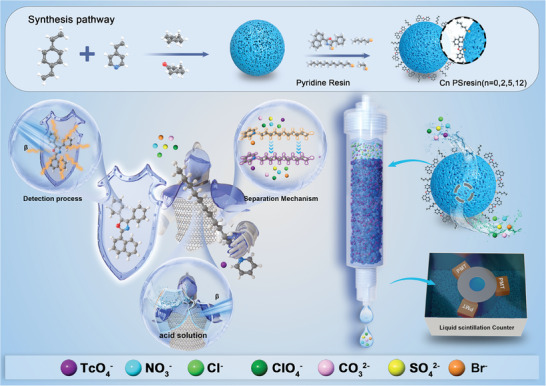
The design ideas, synthesis route, and application diagram of Cn PSresin.

To completely solve the stability and selectivity problems of PSresin for capturing ^99^Tc in complex environments, we proposed a design scheme of double grafting of extractant and fluorophore to obtain a robust PSresin to maintain the ^99^Tc sustained and quick detection. Specifically, as shown in **Figure** **1**, the resin was fabricated by using 4‐vinylpyridine^[^ ^20^ ^]^ as the polymer matrix cross‐linked with divinylbenzene to weave a polymeric armor capable of withstanding extreme environments, such as β‐radiation or acidic solutions. Then the modified BrNPO was grafted onto the pyridine by quaternization, which gave the resin detection ability and ensured the stability of the scintillation group by chemical bonds. Additionally, due to the selectivity of pyridine and the limitation of low acidity, the quaternary amination reagents with different alkyl chain lengths were used to synthesize Cn PSresin (n = 0, 2, 5, 12) to optimize the performance for detecting and capturing the ^99^Tc. The adsorption capacity and kinetics of C2 PSresin are attained surpassing all reported resin materials under the experimental conditions. Even in a high salinity environment of 800 kGy or 3.0 mol L^−1^ HNO_3_, C2 PSresin still maintains high adsorption and detection ability after 10 cycles. The DFT calculations and molecular dynamics (MD) simulations were consistent with the experimental results, which demonstrated that C2 PSresin exhibited superior selective capturing ability and detection efficiency for ^99^TcO_4_^−^ in the presence of excessive anion interference. Therefore, this new robust PSresin exhibits much safer, higher selectivity, and lower cost, offering a novel and highly adaptable approach for the rapid analysis of ^99^Tc, especially in emergency detection scenarios.

## 2 Results and Discussion

### 2.1 Characterization

The synthesis details of PSresin are listed in the section on materials preparation. By varying the ratio of the matrix and organic fluorophore, the porosity, optical transparency, and size of the Cn PSresin (n = 0, 2, 5, 12) were tuned to the optimum. As can be seen from the Scanning electron microscopy (SEM) images of the obtained polymer (Figure S1, Supporting Information), compared with the C0 PSresin, the morphology of C2, C5, and C12 PSresin exhibited almost no change after the introduction of brominated hydrocarbons. The particle size analysis of the synthesized vinylpyridine resins (Figure S2, Supporting Information) showed that all resins had uniform particle sizes and were normally distributed in the range of 100–200 µm, which contributed to higher detection efficiency in subsequent scintillation measurements. The Energy Dispersive Spectrometer (EDS) measurement also further confirms the structural features of the resulting PSresin. The appearance of the Br energy spectrum in Cn PSresin (n = 2, 5, and 12) proved the successful progress of quaternary amination compared to C0 PSresin.

The fluorescence spectra (Figure S3, Supporting Information) showed that all PSresin exhibits a strong peak of 420 nm, which is within the best response range of photomultiplier tube (PMT),^[^ ^21^ ^]^ while the maximum emission wavelengths of 2,5‐Diphenyloxazole (PPO) and 1,4‐bis‐2(5‐phenyloxazoyl)benzene (POPOP) in plastic scintillators are expected to be ≈425 nm. The observed shift to UV light in the peak wavelength can be attributed to the introduction of 2‐(1‐Naphthyl)‐4‐bromo‐5‐phenyloxazole (BrNPO), whose conjugate structure is located between PPO and POPOP, and the lack of secondary fluorescent solute to further transmit the emission spectral wavelength. Since BrNPO was only 3% of the resin matrix mass, the quaternary amination results are focused on discussing in the characterization analysis described below. As can be seen from the BET measurement, The N_2_ adsorption and desorption isotherms of these samples at low relative pressure indicated that the BET (Brunauer−Emmett−Teller) specific surface area and total pore volume of PSresin (Table S1 and Figure S4, Supporting Information) increased after grafting quaternary amination, which was beneficial for TcO_4_^−^/ReO_4_^−^capture due to the increase of adsorption sites. However, the BET‐specific surface area and pore volume gradually decreased with the growth of the quaternary amination carbon chain (specific surface area trend: C2 (63.85 m^2^ g^−1^)> C5 (53.35 m^2^ g^−1^) > C12 (44.72 m^2^ g^−1^), pore volume trend: C2 (0.17 cm^3^ g^−1^) > C5 (0.16 cm^3^ g^−1^) > C12 (0.14 cm^3^ g^−1^)). These results mainly ascribe that the alkyl chains on pyridine nitrogen expand the spatial structure of the polymer backbone, and the longer alkyl chains occupy a small amount of space in the pores.^[^ ^22^ ^]^ The pore size distributions reveal that all the PSresins possessed hierarchically porous structures, and still had a small mesoporous of 18.5 nm after functionalization (Figure S5, Supporting Information). Thermogravimetric Analysis (TGA) was employed to evaluate the thermal stability of Cn PSresin (Figure S6, Supporting Information). As can be seen from the DTG curve the mass loss occurred in three stages, and the weight loss between 50–100 °C was mainly ascribed to the volatillzation of structural water. In the second stage of weight loss ranging from 200 to 400 °C, Cn PSresin (n > 0) has two different weight loss peaks, and the mass loss occurred earlier than C0 PSresin, which is due to the earlier decomposition of the quaternary amine structure in Cn PSresin leading to the loss of the alkyl chain, and then the further decomposition of pyridine molecules. The final mass loss of PSresin was 82%, and its weight loss between 400 ‐800 °C was mainly due to the decomposition of the diethylbenzene resin matrix.

**Figure 2:**
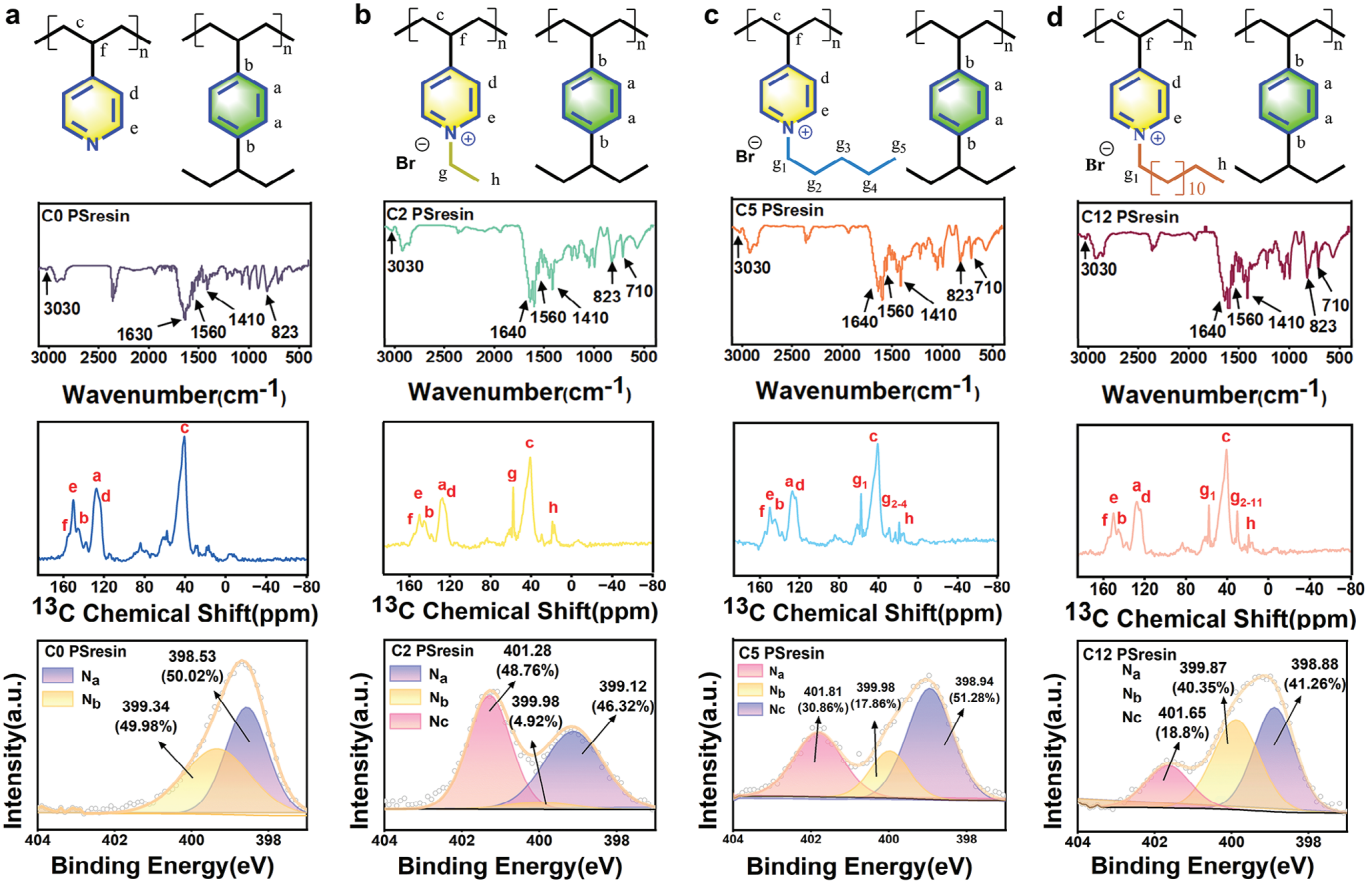
a–d) ^13^C SSNMR spectrum, the FT‐IR spectrum, and XPS spectra of Cn PSresin.

To further verify the resin structure, the ^13^C SSNMR spectra were conducted (**Figure** **2**). The peak at the range of 120∼160 mg L^−1^ was caused by the structure of diethylbenzene and 4‐vinylpyridine matrix. Compared with the pristine matrix, methylene (g) and methyl (h) showed strong peaks at 57.9 and 19.4 mg L^−1^, respectively. Multiple methylenes (g_n_) of alkyl chains in C5 and C12 showed an additional peak at 28.4 mg L^−1^,^[^ ^23^ ^]^ and the peak intensity increased with increased carbon chain length. The ^13^C SSNMR spectra provided direct evidence for the successful modification of Cn PSresin. Similar results were found in FT‐IR spectra, (Figure 2), intrinsic peaks generated by tensile vibrations of the C═C and C═N bonds can be observed at 1630, 1560, and 1410 cm^−1^, confirming the presence of the pyridine ring.^[^ ^24^ ^]^ Moreover, the quaternization of the pyridinyl group split the C═C absorption peak in the benzene ring shift to 1600 cm^−1^, and the absorption peaks of the C═C tensile vibration in the pyridine ring are assigned from 1630 to 1640 cm^−1^, along with the appearance of the CH_2_ bond at 710 cm^−1^.^[^ ^25^ ^]^ It is noteworthy that the appearance of peaks at 3030 and 823 cm^−1^ is due to the stretching and out‐of‐plane bending vibrations of aromatic C─H. The XPS analysis results further confirmed the structural features of PSresin (Figure 2). For the N_1S_ fine spectrum, only two states of N_1s_ are contained in C0 PSresin., pyridyl tertiary amine (N_a_: 398.53 eV) and protonated amine (N_b_: 399.34 eV),^[^ ^20a^ ^]^ respectively. However, Cn PSresin (n > 0) showed a higher binding energy at a peak of near 401.28 eV, which is mainly attributed to the appearance of quaternary amine (Nc) in the pyridine ring.^[^ ^26^ ^]^ Furtherly, the quaternary amine nitrogen content was estimated as 48.76%, 30.86%, and 17.80% for C2, C5, and C12 PSresin (defined as N_c_/(N_a_+N_b_+N_c_)).^[^ ^23b^ ^]^ Evidently, the ethyl group has less steric hindrance and weak electrostatic repulsion, making it more convenient for quaternary amination grafting. However, steric hindrance gradually increases with the chain length increasing, which leads to a difficult grafting process. Therefore, all the results identified the successful synthesis of modified Cn PSresin.

### 2.2 Adsorption Experiment

To compare the effects of different reagents on the adsorption of TcO₄⁻ by PSresin and based on our characterization data, we propose that the modifications may influence the TcO₄⁻/ReO₄⁻ capture efficiency through the following factors: 1) Specific surface area. 2) Grafting ratio. A higher content of quaternary amine nitrogen increases the number of adsorption sites available on the resin. 3) Protonation efficiency of pyridine groups. The resin can utilize the protonation of pyridine nitrogen to form pyridinium ions (Py⁺), which possess anion exchange capabilities. The efficiency of protonation is influenced by the quaternization reagents used and the solution environment. 4) Selectivity of pyridine functional groups toward TcO₄⁻. 5) Electrostatic potential distributions of pyridine molecules. 6) Hydrophobicity of PSResin. Considering that these factors collectively impact adsorption in practical scenarios, adsorption experiments, and theoretical calculations were used to screen the optimal resin.

#### 2.2.1 Adsorption Performance of Re(VII) onto Cn PSresin

**Figure 3:**
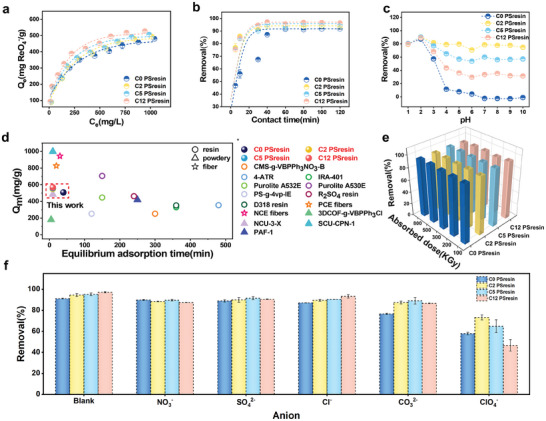
a) Effect of initial concentration on Cn PSresin. b) adsorption kinetics study of Cn PSresin. c) Removal of Cn PSresin at different pH values. d) Comparison of equilibrium adsorption time and maximum adsorption capacity of various reported adsorbents and the references were in Table S2 (Supporting Information). e) adsorption capacities by Cn PSresin after being irradiated with varied doses of β‐rays. f) Effect of competing anions on ReO_4_^−^ removal by Cn PSresin (initially 150 mg L^−1^, mass ratio = 1:10).

Given the absence of other nonradionuclides for ^99^Tc, we conducted batch and dynamic column experiments employing Re as a surrogate for Tc due to their chemical similarities.^[^ ^27^ ^]^ As shown in **Figure** **3**a, the adsorption capacity of PSresin gradually increased and finally approached saturation with the increase of the ReO_4_^−^ concentration. The adsorption behavior fits well with the Langmuir model (*R* ^2^ > 0.98), which is better than that of the Freundlich isotherm model and D‐R model, implying monolayer adsorption process and adsorption energy E > 8 kJ mol^−1^ was dominated by chemical adsorption (**Table** **1**; Figure S7, Supporting Information).^[^ ^28^ ^]^ The maximum adsorption capacity (*q_max_*) of ReO_4_^−^ gradually increased from 507.6 to 568.2 mg g^−1^ with the increase of carbon chain for Cn PSresin. Moreover, the *q_max_* of quaternized PSresin was increased by 50 mg g^−1^ compared with C0 PSresin, which proved that quaternary amination is important to improve the adsorption properties of PSresin. More importantly, the as‐synthesized Cn PSresins demonstrated superior *q_max_* compared to previously reported anion exchange resins, such as Purolite A532E (446 mg g^−1^) and IRA‐401 (330 mg g^−1^), 4‐ATR (354 mg g^−1^), R_2_SO_4_ resin (462 mg g^−1^) (Table S2, Supporting Information), indicating their versatility for Tc capture in a wide range system.

**Table 1:** Isotherm parameters for the adsorption of ReO_4_^−^ on Cn PSresin were calculated by Freundlich, Langmuir, and D‐R models.

PSresin	Langmuir			Fredunlich			D‐R		
	*q_max_* [mg g^−1^]	*K_L_* [L g^−1^]	*R* ^2^	*n^b^*	*K_F_* [mg^1‐n^ L^n^ g^−1^]	*R* ^2^	*K_DR_*	*E* [kJ mol^−1^]	*R^2^*
C0	507.6	0.0091	0.9870	2.905	46.23	0.9514	3.726 × 10^−9^	11.58	0.9681
C2	549.5	0.0072	0.9918	2.920	31.57	0.9513	4.588 × 10^−9^	10.44	0.9729
C5	558.6	0.0077	0.9906	2.510	35.54	0.9633	4.361 × 10^−9^	10.71	0.9772
C12	568.2	0.0108	0.9939	2.703	46.88	0.9494	3.956 × 10^−9^	11.24	0.9735

The adsorption kinetics of Cn PSresin are depicted in Figure 3b. As can be seen the removal ratio of Cn PSresin (n = 2, 5, 12) for ReO_4_^−^ are much faster under equilibrium pressure (< 10 min) than that of C0 PSresin (40 min), which can be attributed to the electrostatic attraction of surface protonated pyridine nitrogen for the tertiary amine adsorption, while the quaternary amine was directly interaction with ReO_4_ by ion exchange. The adsorption kinetics can be followed by the pseudo‐second‐order (*R* ^2^ > 0.99), with C2 PSresin exhibiting a K_2_ value of 1.67 × 10^−2^ g mg^−1^ min^−1^. (Table S3 and Figure S8, Supporting Information). This value exceeds that of the vast majority of reported materials. These results suggested that the adsorption process between Cn PSresin and ReO_4_^−^ was mainly controlled by chemisorption. Remarkably, the adsorption rate of Cn PSresin for ReO_4_^−^ is also much faster than that of most reported sorbents (Figure 3d), and the high adsorption capacity and fast kinetic response of Cn PSresin make it more attractive in treating high‐level radioactive waste containing ^99^Tc compared with pure inorganic materials,^[^ ^29^ ^]^ resin,^[^ ^30^ ^]^ MOF,^[^ ^31^ ^]^ COF.^[^ ^2b^ ^]^

To further investigate the applicability of the PSresin to capture ReO_4_^−^ in different solutions, the effect of pH on the adsorption was conducted (Figure 3c). The adsorption capacity of the original C0 PSresin decreased significantly when the pH value was over 3, while C2 PSresin remained near the saturated adsorption capacity within the measured pH range (1–10) due to its high grafting rate. The Zeta potential showed the same trend (Figure S9, Supporting Information). When pH>4, the protonation of pyridine nitrogen is inhibited by a higher concentration of OH^−^, while the quaternary aminating resin presents a positive Zeta potential when pH = 1–10, which facilitates anion exchange. Combined with XPS results analysis, C2 PSresin has the widest application range and owns the best performance for ReO_4_^−^ adsorption. Given that actual analysis samples, such as spent fuel, are usually in nitric acid, the adsorption properties of C2 PSresin were further explored in a 3.0 mol L^−1^ HNO_3_ system by varying the solid–liquid ratio (Figure S10, Supporting Information). Even in 3.0 mol L^−1^ HNO_3_, 40 g L^−1^ C2 PSresin can reach an adsorption ratio of more than 70% for 150 mg L^−1^ Re under experimental conditions, which is more than enough to meet the requirements of nitric acid system samples in column experiments.

#### 2.2.2 Irradiation Stability

The radiation resistance of Cn PSresin is another important factor for application since there may be potentially strong radiation in the collected samples. As shown in Figure 3e, the β ray radiation properties of the Cn PSresin were evaluated at different radiation doses (100, 200, 300, 500, 800 kGy). Even at a radiation dose of 800 KGy, the adsorption properties of the Cn PSresin were maintained well, indicating, the good radiation resistance of Cn PSresin. The FT‐IR spectra also showed that the vibrational properties of Cn PSresin changed negligibly after exposure to high doses of β‐rays, indicating the stability of key functional groups (Figure S11, Supporting Information). This is because all the quaternary amine sites are located in the polymer chain and have rich conjugate ring properties. When exposed to external radiation, the key functional groups are protected by a large conjugate system and are difficult to degrade by radiation.

#### 2.2.3 Effect of Competing Ions on ReO_4_^−^ Adsorption

Considering the properties of TcO_4_^−^ as a monovalent oxygen‐containing anion, the interferences of other anions, including NO_3_^−^, Cl^−^, SO_4_^2−^, ClO_4_^−^ and CO_3_^2−^ were employed to evaluate the anti‐interference ability of Cn PSresin. (Figure 3f; Figure S12, Supporting Information). To exclude the case that the adsorption sites are not fully occupied, all experiments were carried out in the presence of ReO_4_^−^ and interference ions at higher absolute values and ratios. Overall, the removal efficiencies of all Cn PSresins toward ReO_4_^−^ were negligibly influenced at the mass ratios of 1 and 10 (interfering ions/ReO_4_^−^). However, the selectivity of Cn PSresin at higher concentrations of Cl^−^, CO_3_^2−^, and ClO_4_^−^ was greatly improved after quaternization, among which C2 PSresin had the best performance, with adsorption ratio exceeding 80% under most conditions. In particular, when the mass ratio of NO_3_^−^: ReO_4_^−^ was 1000:1 (the molar ratio is ≈4036:1), the adsorption ratio of C2 PSresin is better than that of most reported COF materials. ([C_2_vimBr]_24%_‐TbDa‐COF^[^ ^32^ ^]^ (50% at the molar ratio of 100:1), CPN‐tpm^[^ ^33^ ^]^ (≈30% at the molar ratio = 1000:1) and SCU‐CPN‐1^[^ ^34^ ^]^ (50% at a molar ratio of 1000:1) Moreover, the adsorption ratio of C2 PSresin was maintained at 90% when the mass ratio of SO_4_^2−^: ReO_4_^−^ was 1000:1 (molar ratio of 2606:1). The better selective adsorption ability makes C2 PSresin more advantageous in the treatment of real samples, enabling it to enrich TcO_4_^−^ selectively from unknown complex systems for further measurement.

#### 2.2.4 Desorption and Reusability

The spent adsorbent was used to investigate the regeneration capability of C2 PSresin by employing seven desorption agents. The results demonstrated that ReO_4_^−^ can be easily desorbed through C2 PSresin by using 2.0 mol L^−1^ HNO_3_, NaNO_3,_ and NaClO_4_ (Figure S13, Supporting Information). Considering HNO_3_ has the best desorption effect, 2.0 mol L^−1^ HNO_3_ was used as the desorption agent for reusability subsequently. Importantly, C2 PSresin showed excellent reusability, of which the adsorption ratio was higher than 90% and the desorption efficiency was higher than 95% in the ten adsorption‐desorption cycles (Figure S14, Supporting Information).

### 2.3 Theoretical Calculation

**Figure 4:**
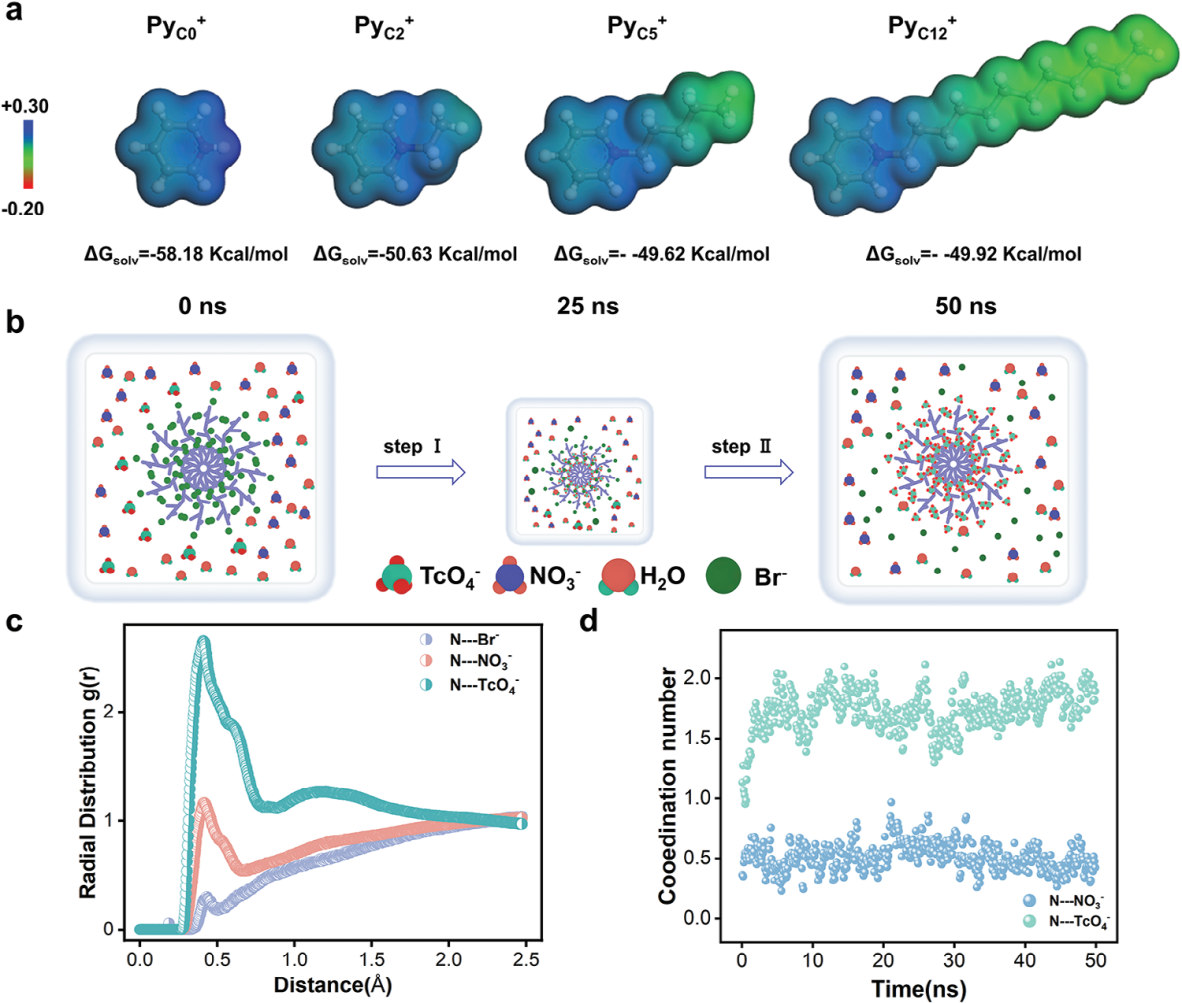
a) Electrostatic potential (ESP) distributions on the van der Waals surfaces (isodensity = 0.017 a.u.) of Py_Cn_^+^. b) Selected snapshots from the molecular dynamic simulation showing the anion exchange process (TcO_4_^‐^ replacing Cl^−^) on C2 PSresin in HNO_3_ solution. c) Radial distribution functions of the anion around the Pyridine nitrogen at 49 ns. d) Coordination number of NO_3_^−^, TcO_4_^−^ with nitrogen atoms.

To verify the superior selectivity adsorption of Cn PSresin for ReO_4_^−^/TcO_4_^‐^, DFT calculations were used to reveal the intrinsic driving force for its adsorption capacity. The interaction between *N*‐quaternary pyridine fragment (Py_cn_^+^) of different alkyl chains and ReO_4_^−^/TcO_4_^−^ was comprehensively conducted, and synchronously compared with Cl^−^, NO_3_^−^ and ClO_4_^−^. As shown in **Figure** **4**a, the positive ESP (blue areas) mainly distributes on the vdW surface of pyridine N, and the carbon chains of C5 and C12 make minimal contributions to the positive ESP. Furthermore, the calculated solvation energy (ΔGsolv) indicated that the hydrophobicity of the M^+^ fragment could be effectively improved by installing alkyl chains since pyridine and alkyl chains are typical hydrophobic functional groups. The enhanced hydrophobicity is likely to provide a theoretical basis for the increased selectivity of PSresin because the hydration standard of ReO_4_^‐^ (−330 kJ mol^−1^) is much lower than SO_4_^2−^ (−1090 kJ mol^−1^)^[^ ^35^ ^]^ and like NO_3_^−^ (−306 kJ mol^−1^). In addition, it is worth nothing that ReO_4_^−^ and TcO_4_^−^ appear large volumes and low overall charge density, increasing the hydrophobicity of PSresin is more conducive to the ion exchange process in accordance with the principle of anti‐Hofmeister bias.^[^ ^36^ ^]^ However, the long alkyl chain may cause steric hindrance effects. Overall, the cooperation of the hydrophodicity enhancement and the additional steric stability brought by the appropriate introduction of hydrophobic hydrocarbon chains on pyridine effectively improved the selectivity for ReO_4_^‐^ and TcO_4_^−^.

Compared with the approved experimental results, the structure of Py_Cn_^+^(Br^−^) and after ion exchange with each anion was optimized and the *Δ*E values were calculated. As Figure S15 (Supporting Information) showed, only the *Δ*E value of the ion exchange between Py_Cn_^+^(Br^−^) and Py^+^(ReO_4_^−^/TcO_4_^−^) in C2 PSresin is negative, indicating that the C2 fragment is energetically more likely to bind to ReO_4_^−^/TcO_4_^−^ than the other configurations. Moreover, compared with the Co fragment, Py_Cn_^+^(Br^−^) → Py_Cn_^+^(Cl^−^) becomes more and more difficult to occur with the extension of an alkyl chain, which is consistent with the trend shown in the experimental results. The longer the alkyl chain, the better the selectivity for Cl^−^. On the contrary, the C2 fragment is more susceptible to NO_3_^−^, hence the removal rate of C2 PSresin is reduced more than that of C0 PSresin in the batch experiments of high concentrations of NO_3_^−^. Consistent with the experimental results, C2 PSresin exhibited the best overall selectivity. This may be because the pyridine functional group in Cn PSresin is already attached to the polymer chain, and the excessive hydrophobic interactions introduced by longer alkyl chains make the resin less selective compared to C2 PSresin.

Further, to more accurately simulate the actual adsorption situation, we explored the adsorption dynamics of TcO_4_^−^ on C2 PSresin in HNO_3_ solutions by using molecular dynamics (MD) simulations. At the beginning of the ion exchange process, Br^−^ anions are located at the pyridine‐N^+^ sites (Figure 4b). Subsequently, ion exchange between TcO_4_^−^ and Br^−^ occurred in a very short time, and C2 PSresin also showed a significant binding affinity for TcO_4_^−^ at high concentrations of NO_3_^−^ at 50 ns. After ion exchange, the results of the Radial Distribution Function (RDF) for C2 PSresin are depicted in Figure 4c, at 49 ns, the amount of TcO_4_^−^ per unit distance from pyridine nitrogen is higher, while Br^−^ is exchanged to a longer distance. Furthermore, the coordination number between acid ions and N^+^ was further derived from RDF (Figure 4d), compared with NO_3_^−^, TcO_4_^−^ exhibits a stronger attraction to pyridine nitrogen. The above results further verified the efficient adsorption ability of C2 PSresin to TcO_4_^−^ in the nitric acid environment in the actual experiment.

### 2.4 Scintillation Properties and Radiation Detection

To quantify measurement properties of scintillation for Cn PSresin, we quantitatively measured the detection efficiency of ionizing radiation after adsorption. The detection efficiency of Cn PSresin was measured using 10 mL of 800 Bq L^−1^ Tc(VII) solution (in 0.1 mol L^−1^ HCl), all adsorption efficiencies were more than 98%, and the total detection efficiency is shown in Table S4 (Supporting Information) (The experimental setup is shown in Figure S16, Supporting Information). As a result, the detection efficiency gradually decreases as the number of carbon chains used for quaternization increases, which was caused by the blockage of pore structure by longer alkyl chains and steric hindrance between alkyl chains and BrNPO in the one‐pot cooking reaction. In conclusion, C2 PSresin has better adsorption properties than other Cn PSresins, so the C2 PSresin was selected for subsequent experiments. In order to explore the detection limit of PSresin, a 1.2 g blank sample of fresh Cn PSresin was measured by column experiment condition, 10 mL 0.1 mol L^−1^ HCl was used as a reference solution and four groups were parallel to obtain blank counts. The minimum detectable concentration (MDC) could be estimated using the Currie Equation (1):^[^ ^37^ ^]^

(1) $$MDA\left(\mathrm{Bq}\right)=\frac{k^{2}+2k\sqrt{2N_{B}}}{T_{\varepsilon }}\;$$

where *k* = 1.645 for a 95% confidence level; *N_B_* is the background count; *T* is the counting time (s); *ε* is the counting efficiency shown above. Based on the measurement of 4 blank samples, the average MDA of the four scintillation resins is lower than 0.03 Bq for 10 mL ^99^Tc solution with 3600 s of counting time. It is proved that PSresin can be used in the capture and detection of Tc solution with low activity.

**Figure 5:**
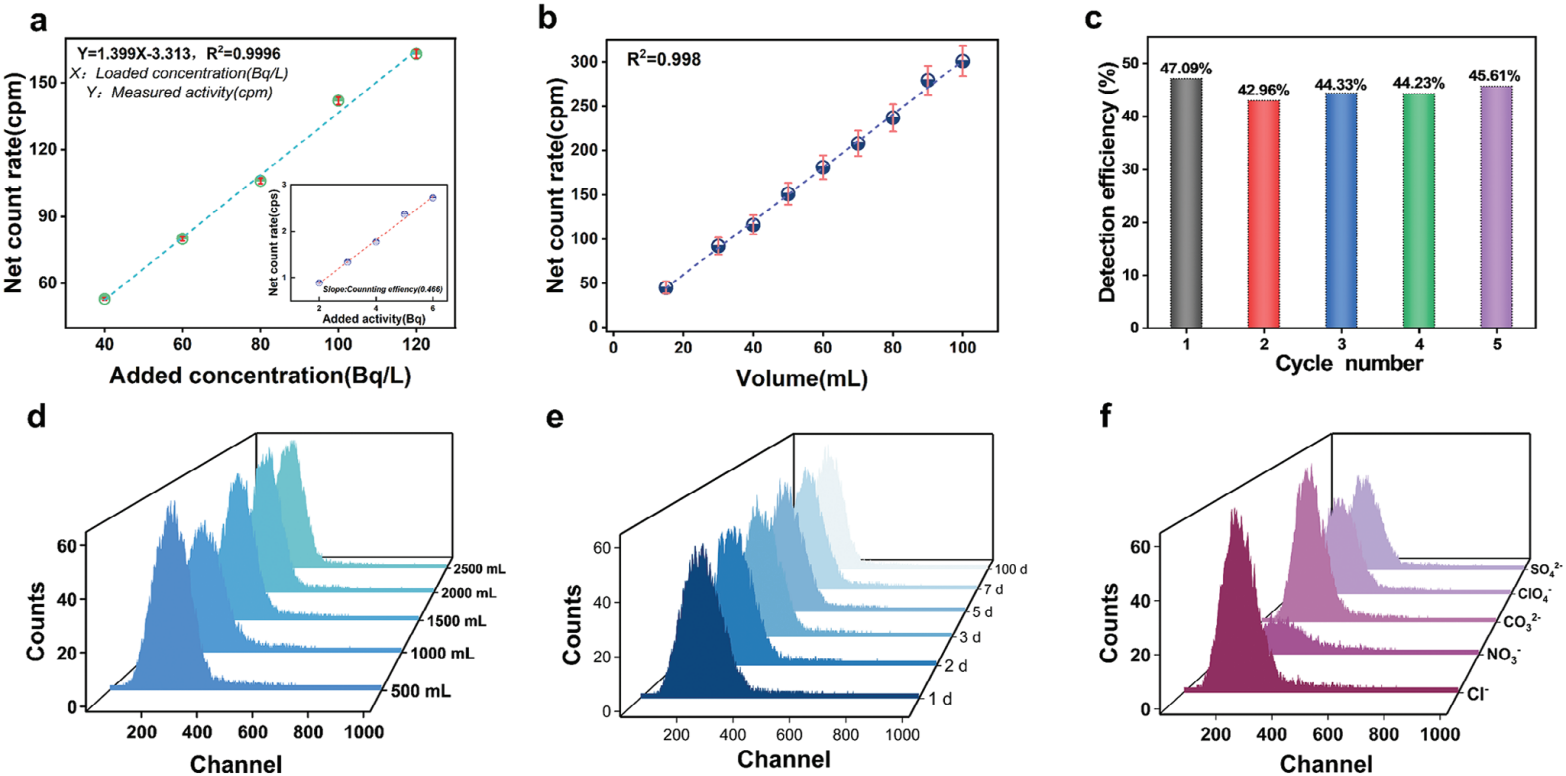
a) The calibration curves of C2 PSresin. b) Continuous measurements of ^99^Tc by C2 PSresin. c) C2 PSresin multiplexed scintillation performance. d) Effect of pretreatment on scintillation performance using 500–2500 mL of 0.1 mol L^−1^ HNO_3_ solution e) Effect of time stability on spectra. f) Spectra of C2 PSresin with 2 mol L^−1^ of different anions were measured. The count time was 60 min for each standard.

C2 PSresin showed the best comprehensive capture performance, and the detection efficiency was close to that of the quaternary aminating sample. Therefore, C2 PSresin was used to establish a standard curve to verify the detection method, which can be used to measure the sample concentration for offline detection. The calibration curves were established by adding 10 mL of different concentrations of Tc(VII) solution as shown in **Figure** **5**a The Tc removal rate of C2 PSresin was higher than 99.5% in all samples, and the final detection efficiency can be determined directly from the slope of calibration curves to be 46.61%. Moreover, the established calibration curves had good linearity (*R* ^2^ > 0.999), which could meet the needs of Tc(VII) analysis in this concentration range.

When analyzing the concentration of Tc in spent fuel reprocessing solutions, a rapid detection mode with continuous measurement is essential. Therefore, the stability of detection efficiency through continuous testing in the solution is also a crucial metric for PSresin. As shown in Figure 5b, C2 PSresin was used for continuous measurements of a ^99^Tc solution at 120 Bq L^−1^. The count of Tc had a linear relationship with the volume of solution (*R* ^2^ = 0.998), and the PSresin can be used for continuous analysis and detection of Tc. The impact of repeated use on the detection efficiency of C2 PSresin was further evaluated. As shown in Figure 5c, the detection efficiency of the scintillation resin remained stable throughout five cycles of reuse, highlighting the success of our design concept and the exceptional stability of C2 PSresin. Additionally, PSresin was pretreated with 500–2500 mL of HNO_3_ solution to assess whether organic fluorophore shedding occurred or nitric acid‐induced further oxidative yellow, which could potentially diminish detection efficiency. As indicated in Figure 5d, the scintillation resin spectra remained consistent up to 2500 mL of solution. These results confirm the accuracy of C2 PSresin in counting ^99^Tc across various application scenarios. The standard spectrum of ^99^Tc measured by scintillation liquid lies from 0 to 600 channel number,^[^ ^38^ ^]^ and the C2 PSresn pulse height spectrum was shifted to lower channel number, probably caused by the loss of signal transmission energy on the solid and the quenching of the resin matrix.^[^ ^19b^ ^]^ Figure 5e presents the effect of time stability on spectra, considering storage times of 1, 2, 3, 5, 7, and even 100 days, respectively. The results reveal that the spectra of samples with storage time up to 100 d exhibit negligible variation, demonstrating high consistency and stability, thereby confirming the accuracy of the count within a specific storage period after combining with ^99^Tc. Coupled with continuous measurement data, C2 PSresin has proven to be highly suitable for long‐term continuous analysis in automated systems.

The pulse height spectrum of C2 PSresin for 8 Bq ^99^Tc was also measured with various 2 mol L^−1^ anion as interferences (Figure 5f). The adsorption efficiency for all columns exceeded 98% except NO_3_^−^. With a counting time of 3600 s, the spectra showed that measurements for Cl^−^, CO_3_^2−^, SO_4_^2−^, and ClO_4_^−^ corresponded accurately with the presence of ^99^Tc. The Cl^−^ and CO_3_^2−^ samples exhibited narrower peaks and lower energies, while the spectra for ClO_4_^−^ and SO_4_^2−^ appeared in the same position. In the HNO_3_ medium, deviations in measurement efficiency may be attributed not only to changes in the adsorption rate but also to the potential oxidation of the pyridine resin by nitric acid, which can lead to color quenching and a consequent reduction in measurement efficiency.

## 3 Conclusion

In this work, we developed a stable plastic scintillation resin (Cn PSresin) via suspension polymerization and chemical grafting, utilizing 4‐vinylpyridine and divinylbenzene as matrix components. Experimental results and theoretical calculations confirmed that C2 PSresin has superior comprehensive performance with a maximum adsorption capacity of 549.2 mg g^−1^ for ReO_4_^−^ in a mere 10 min. Remarkably, it demonstrates exceptional structural robustness and remarkable selectivity far beyond similar materials even under harsh conditions, retaining consistent adsorption properties upon ten cycles. C2 PSresin also exhibited exceptional sensitivity with the minimum detectable concentration of 0.0180 Bq and a detection efficiency of 44.17±1.86%. Furthermore, continuous measurements reveal an average detection efficiency of 46.61%. Therefore, this new robust PSresin exhibits much safer, higher selectivity, and lower cost, offering a novel and highly adaptable approach for the rapid analysis of ^99^Tc, especially in emergency detection scenarios.

## 4 Experimental Section

### Chemicals and Materials

4‐Vinylpyridine (96%, Contains 80−120 mg L^−1^ hydroquinone), Divinylbenzene (80%), Gelatin (99%), CaCO_3_ (99%), 2,2'‐Azobis(2‐methylpropionitrile) (AIBN) (99%), 2‐(1‐naphthalenyl)‐5‐phenyl‐oxazol (NPO) (99%), purchased from Shanghai Aladdin Biochemical Technology Co., Ltd. Cyclohexanone (99%), Cyclohexane (AR, 99.7%), *N*‐Bromosuccinimide (AR, 99%), Bromoethane (99%), 1‐Bromopentane (GR, 99%), 1‐Bromododecane (98%) were all purchased from Shanghai Macklin Biochemical CO., Ltd. Methylbenzene (AR, 99.7%) was purchased from Xilong Scientific Co., Ltd. NH_4_ReO_4_ was purchased from Zhuzhou Golden Rhenium Industrial Co., Ltd. The standard solution of ^99^Tc (in the form of NH_4_TcO_4_) was provided by the China Institute of Atomic Energy. Ultrapure water (18.2 MΩ×cm) was used for the preparation of various solutions throughout the experiments. All reactions were carried out under the protection of a nitrogen atmosphere. All chemical reagents were used without additional treatment.

### Materials Preparation—Preparation of Vinylpyridine Resin

In a typical synthesis procedure, Styrene monomer, divinylbenzene crosslinker, AIBN initiator, cyclohexane, and cyclohexanone pore‐making agent were mixed to form a dispersed oil phase. The oil phase was sonicated for 20 min to ensure uniform dispersal. Then mix the H_2_O gelatin and CaCO_3_ into a three‐nicked flask and stir the mixture at 60 °C until the gelatin was completely dissolved. After that, max the oil phase with the aqueous phase. The sealed mixture was stirred at 280 rpm at 70 °C for 0.5 h, at 80 °C for 4 h, and then at 90 °C for 2 h. The resin was collected from the bottom of the flask, which was isolated by filtration and washed with warm water (40–60 °C), acetone, and ultrapure water three times separately and vacuum‐dried at 50 °C for 48 h.

### Materials Preparation—Synthesis of BrNPO

NPO (5.00 g, 18.5 mmol) and *N*‐Bromosuccinimide (3.93 g, 22.2 mmol) were mixed with 100 mL chloroform containing a few drops of hydrobromic acid and stirred at 65 °C for 4 h. The product was cooled to room temperature (25 °C) and the solvent was dried. After that, the product was mixed with 50 mL methanol and cooled to obtain the final product (BrNPO), which was recrystallized, washed, and dried at lowered pressure.^[^ ^19b^ ^]^

### Materials Preparation—Preparation of Cn‐Vinylpridine PSresin (n = 0, 2, 5, 12)

For the preparation process of quaternary amine resin, 5 g of vinylpyridine resin was added into a flask with 100 mL chloroform and BrNPO (0.15 g, 3%wt). Then bromoethane (0.05 mol), 1‐bromoethane (0.05 mol), and 1‐bromododecane (0.05 mol) were added respectively to synthesize different resins. The mixture was ultra‐sounded at 60 °C for 6 h, filtered, and vacuum dried at 50 °C for 48 h. After that, the C0, C2, C5, C12‐Vinylpridine PSresin (Cn PSresin) were obtained (Figure 1).

### Characterization of Cn PSresin

Scanning electron microscopy (SEM, S‐4800, Hiachi, Japan), Energy Dispersive Spectrometer (EDS), Dinitrogen adsorption‐desorption isotherms, Brunauer–Emmett–Teller (BET, ASAP2020M&TriStar3020, USA) method, Fourier Transform Infrared Spectroscopy (FT‐IR, Nicolet Avatar 360, Thermo Nicolet, USA), Thermogravimetry Analysis (TGA, Linseis, Germany), Solid State Nuclear Magnetic Resonance (SSNMR, AVANCE NEO 600MHz WB FT‐NMR, Switzerland), X‐ray Photoelectron Spectroscopy (XPS, AXIS Ultra DLD, Kratos, UK) and Fluor spectrophotometer (LS55, Cernet Corporation, China) were used to characterize the structure of the samples. The zeta potential values at various pH values were determined using a Zeta potential and laser particle size analyzer (Zetasizer Pro, Malvern Panalytical, UK). Detailed test parameters for the various instruments were listed in the Supporting Information.

The radiation‐resistance property of PSresin was evaluated by determining the adsorption performance of irradiated samples at different doses (100, 200, 300, 500, and 800 kGy). The ionizing radiation test was performed by using a β‐ray irradiator (electron accelerator with a dose rate of 14 KGy s‐1, Lanzhou lvyuan Radiation Co., ltd.). The Liquid Scintillation Counter (LSC, Hidex 300 SL Super Low Level, Finland) was used for measurement.

### Batch Adsorption Experiments

In a typical adsorption procedure, 10 mg of Cn PSresin was added to 5 mL aqueous solutions containing 150 mg L^−1^ of Re(VII), and the pH of mixed liquor was adjusted using negligible volume NaOH and HNO_3_ solution. The system was shaken overnight at 25 °C and reached equilibrium, the suspension was separated with a 0.22 µm membrane filter and the concentration of Re(VII) solution was determined by ICP‐OES (PQ9000, Germany). The details regarding the adsorption and desorption experiments can be found in the supplemental information. The adsorption capacity (*q_e_*) and adsorption ratio (*E*%) were respectively calculated by Equations (2) and (3).

(2) $$q_{e}=\frac{C_{0}-C_{e}}{m}\;\times V$$

(3) $$E=\frac{C_{0}-C_{e}}{C_{0}}\;\times 100\%$$

where *C_0_* (mg L^−1^) and *C_e_* (mg L^−1^) were the initial and equilibrium concentration of Re, respectively, *V* (mL) was the solution volume, and *m* (mg) was the solid mass. All the experiments were performed for three duplicates, and the resulting error was evaluated as less than 5%.

### DFT Calculations

The DFT calculation was performed with the Dmol3 package in Material Studio (MS) with functional GGA‐PW91.^[^ ^39^ ^]^ In the geometry optimization and energy calculation process, the convergence tolerances of energy and max displacement were 1 × 10^−6^ Ha and 5 × 10^−3^ Å, respectively. In addition to explicit the solvation effect, the COSMO model was also applied in all ion calculations. The quaternary aminopyridine fragment (Py_cn_^+^ for short in the following) was used as a model to simulate the positively charged local structure of Cn PSresin. The binding energies ΔE of Py_cn_^+^A^–^ (A^–^ = TcO_4_^−^, ReO_4_^−^, Br^−^, NO_3_^−^, Cl^−^) complex formed by five anions was calculated.^[^ ^40^ ^]^ The ΔE was calculated as:

(4) $$\Delta E=E\left(Py_{cn}^{+}A^{-}\right)-E\left(Py_{cn}^{+}\right)-E\left(A^{-}\right)$$

### Separation Procedure and LSC Measurement

For the separation process, 1.2 g of PSresin was placed into a 2 mL column tube. A peristaltic pump was used to pass 10 mL of 0.1 mol L^−1^ HCl through the column for preprocessing, followed by a Tc(VII) solution for adsorption. The column and container were then washed with 10 mL of water. Finally, the resin column was drained using a pump. The collected liquid was analyzed by ICP‐MS. The column tube was placed into a 20 mL scintillation vial for LSC determination. All samples were kept in the dark for 24 h before counting, and LSC measurements were conducted for 1 h.

Continuous measurements were performed with reference to previous work: 1.2 g of Cn PSresin was selected into the column vial, which was designed and made in the laboratory.^[^ ^41^ ^]^ During operation, the solution to be tested was injected into the column experiments, and after the adsorption was completed, the whole device was placed in the LSC. The sample was then repeated and measured every 10 mL of Tc solution to determine PSresin continuous measurement capability. The effluent solution was measured by ICP‐MS to obtain the column efficiency. All the measurements were conducted for 1 h, resulting in less than 5% uncertainty based solely on counting statistics of the individual PSresin.

Scintillation measurements were performed with Hidex 300 SL Super Low Level, which uses advanced triple PMT detector technology to achieve extremely high counting efficiency, and no luminescence counting mode using the triple‐to‐double coincidence ratio (TDCR) method.^[^ ^42^ ^]^

## Conflict of Interest

The authors declare no conflict of interest.

## Author Contributions

T.L. Y.H. and J.Y. contributed equally to this work. T.L. Performed supervision, project administration, formal analysis, conceptualization and wrote reviewed and edited the final manuscript. Y.H. Performed methodology, investigation, formal analysis, data curation, and wrote the final manuscript. J.Y. Performed visualization, project administration, investigation wrote reviewed and edited the final manuscript. K.H. performed investigation, formal analysis, data curation, and wrote the final manuscript. Y.Z. performed methodology. J.T. performed Methodology, Investigation. M.W. performed methodology, investigation. L.H. performed investigation, formal analysis, conceptualization, and wrote reviewed and edited the final manuscript. K.S. performed investigation, funding acquisition, conceptualization, and wrote reviewed and edited the final manuscript. X.H. performed supervision, investigation.

## Supporting information



Supporting Information

## Data Availability

The data that support the findings of this study are available from the corresponding author upon reasonable request.
