# Supplementary material for: Recyclable Robust Plastic Scintillation Resin Achieving the Exceptional Separation and Detection of Technetium‐99
Source: Adv Sci (Weinh). 2024 Nov 5;12(1):2411523. doi: 10.1002/advs.202411523 (PMC11714228; doi:10.1002/advs.202411523)
Supplement: Supplementary file 1 — Supporting Information [file ADVS-12-2411523-s001.docx]

Supporting Information

Recyclable Robust Plastic Scintillation Resin Achieving the Exceptional Separation and Detection of Technetium-99

Tonghuan Liu^†,1^, Yichen Hu^†,1^, Junqiang Yang^†,1^, Kesheng Hu^1^, Bei Qi^2^, Yun Zhou^,1^, Juan Tong^1^, Man Wang^1^, Liang Huang^2*^, Keliang Shi^1*^, Xiaolin Hou^1,2*^

Characterization techniques

The morphologies of the samples were characterized using scanning electron microscopy (SEM). Energy Dispersive Spectrometer (EDS) was used to analyze the distribution of C, N, O, Br on the sample surface. N_2_ adsorption/desorption curves were obtained using an automatic gas adsorption analyzer. The functional groups in the material were analyzed by Fourier transform infrared spectroscopy (FTIR) with a scanning resolution of 2 cm^-1^ and a scanning range of 4000 - 400 cm^-1^. Thermogravimetric analysis (TGA) was conducted using a thermogravimetric analyzer with a temperature range of 25 - 800 ℃ and an airflow heating rate of 10 ℃ min^-1^. X-ray photoelectron spectroscopy (XPS) was carried out using a monochromatic Al Kα X-ray source (1486.6 eV) and the operating conditions are 15 kV and 20 mA. The excitation wavelength of the fluorescence spectrophotometer was 365 nm and the scanning speed was 300 nm min^-1^. The surface zeta potential values of samples at different pH values were measured by zeta potential and particle size analyzer.

**Batch experiments**

Adsorption isotherm investigations. The Adsorption isotherm experiments of PSresin for ReO_4_^–^ were carried out by mixing 10 mg of PSresin with 5 mL of a certain concentration (200 mg L^-1^, 400 mg L^-1^, 600 mg L^-1^, 800 mg L^-1^, 1000 mg L^-1^, 1200 mg L^-1^, 1400 mg L^-1^, 1600 mg L^-1^, 1800 mg L^-1^, 2000 mg L^-1^) of initial Re solution in a glass beaker, the samples were separated and analyzed after being stirred for 24 hours.

Two isotherm models, including Langmuir isotherm model (1) and Freundlich model (2) were used to fit the sorption data and can be expressed in the following equations:

Langmuir model:

| $\frac{\boldsymbol{C}_{\boldsymbol{e}}}{\boldsymbol{q}_{\boldsymbol{e}}}\boldsymbol{=}\frac{\boldsymbol{1}}{\boldsymbol{q}_{\boldsymbol{m}}\boldsymbol{K}_{\boldsymbol{L}}}\boldsymbol{+}\frac{\boldsymbol{C}_{\boldsymbol{e}}}{\boldsymbol{q}_{\boldsymbol{m}}}$ | **(1)** |
| --- | --- |

Freundlich model:

| $\boldsymbol{ln}\boldsymbol{q}_{\boldsymbol{e}}\boldsymbol{=ln}\boldsymbol{K}_{\boldsymbol{F}}\boldsymbol{+}\frac{\boldsymbol{1}}{\boldsymbol{n}}\boldsymbol{ln}\boldsymbol{C}_{\boldsymbol{e}}$ | **(2)** |
| --- | --- |

where q_m_ (mg g^-1^) is the maximum sorption capacity of ReO_4_^–^, *K_L_*, *K_F_*, and *n* are the Langmuir constants, Freundlich constants and the sorption intensity, respectively.

Dubinin-Radushkevich (D-R) model:

| $\boldsymbol{ln}\boldsymbol{q}_{\boldsymbol{e}}\boldsymbol{=ln}\boldsymbol{q}_{\boldsymbol{max}}\boldsymbol{-}\boldsymbol{K}_{\boldsymbol{DR}}\boldsymbol{\varepsilon}^{\boldsymbol{2}}$ | **(3)** |
| --- | --- |
| $\boldsymbol{\varepsilon=RTln(1+}\frac{\boldsymbol{1}}{\boldsymbol{C}_{\boldsymbol{e}}}\boldsymbol{)}$ | **(4)** |
| $\boldsymbol{E=}\frac{\boldsymbol{1}}{\sqrt{\boldsymbol{2}\boldsymbol{K}_{\boldsymbol{DR}}}}$ | **(5)** |

where *qe* (mol g^-1^) and *Ce* (mol L^-1^) represent the molar equilibrium adsorption amounts and concentrations, respectively. *K_DR_* is the equilibrium constant, *ε* is the Polanyi potential, *T* (K) is the Kelvin temperature, *R* (J mol^-1^ K^-1^) is the ideal gas constant, and *E* (kJ mol^-1^) is the adsorption energy. In the D-R isotherm model, the type of adsorption reaction can be determined by the adsorption energy E: when E < 8 kJ mol^-1^, it is physical adsorption, and when E > 8 kJ mol^-1^, it is chemical adsorption.

For the adsorption kinetics study, 10 mg PSresin was mixed with 5 mL solution containing 150 mg L^-1^ ReO_4_^–^, the system contained 0.1mol L^-1^ HNO_3_, Fully shocks a certain time (5 min, 10 min, 30 min, 40 min, 60 min, 80 min, 100 min, 120 min) quickly after using 0.22 μm membrane separation. The Pseudo-first-order and Pseudo-second-order model were used to analyze sorption kinetics. The two models are expressed as followed:

Pseudo-first-order model:

| $\boldsymbol{ln}\left( \boldsymbol{q}_{\boldsymbol{e}}\boldsymbol{-}\boldsymbol{q}_{\boldsymbol{t}} \right)\boldsymbol{=ln}\boldsymbol{q}_{\boldsymbol{e}}\boldsymbol{-}\boldsymbol{k}_{\boldsymbol{1}}\boldsymbol{t}$ | **(6)** |
| --- | --- |

Pseudo-second-order model:

| $\frac{\boldsymbol{t}}{\boldsymbol{q}_{\boldsymbol{t}}}\boldsymbol{=}\frac{\boldsymbol{1}}{\boldsymbol{k}_{\boldsymbol{2}}\boldsymbol{q}_{\boldsymbol{e}}^{\boldsymbol{2}}}\boldsymbol{+}\frac{\boldsymbol{t}}{\boldsymbol{q}_{\boldsymbol{e}}}$ | **(7)** |
| --- | --- |

Where *k_1_* (min^-1^) and *k_2_* (g mg^-1^ min^-1^) are constants of Pseudo-first-order model and pseudo-second-order model, respectively. qt and qe are the sorption capacity at time t and equilibrium time, respectively.

Effect of pH. The effect of pH for ReO_4_^–^ adsorption of PSresin was evaluated by adding 10 mg of PSresin to 5 mL of 150 mg L^-1^ Re solution, and subsequently the pH was adjusted to 1-10 within 5 minutes using high concentrations of HNO_3_ and NaOH, respectively. The samples were separated and analyzed after stirred for 24 hours.

Anion selectivity studies. The effect of excessive competitive ions was determined by loading 10 mg of PSresin into 5 mL of mixed solution containing 150 mg L^-1^ of Re, then adding a certain mass concentration of competitive anions (NO_3_^–^, SO_4_^2–^, Cl^–^, CO_3_^2–^and ClO_4_^–^), and finally making Re: anion=1:1, 1:10, 1:100, 1:1000. The samples were shaken thoroughly for 24 hours and then separated by filter membrane for further measurement.

Irradiation stability measurement. In this section, the adsorption capacity of irradiated PSresin was evaluated. The PSresin was irradiated in β-rays to obtain the irradiated PSresin material. Then 10 mg of irradiated psresin was mixed with 5 mL of 150 mg L^-1^ Re solution, and the mixture was shaken thoroughly for 24 hours before separation and analysis.

Desorption experiments. Firstly, 50 mg PSresin was added to 25 mL mixed solution containing 150 mg L^-1^ Re, and the supernatant was removed by centrifugation after full shaking. The PSresin was dried at 50 ℃, the Re content in the supernatant was measured, and the adsorption capacity of PSresin was calculated. Then 10 mg of the adsorbed PSresin was weighed and added to 5 mL of the resolving agent solution (containing 2 mol L^-1^ NH_3_H_2_O, NaOH, NaClO_4_, NaNO_3_, HNO_3_, NaCl, HCl respectively), and the solution was separated and measured after full agitation for 24 hours.

**Molecular dynamics simulation methods**

Classical molecular dynamics (MD) simulations were used to investigate the interaction of Cl^–^ and TcO_4_^–^ with C2 PSresin at the atomic level under alternating electric fields. A random polymer of polymerization degree 10 was constructed using a 1:1 ratio of C_8_H_10_NBr and diethylbenzene. Then the polymer molecules were constructed using 20 such polymer chains, and the assurance system contains 100 quaternary amine sites. The initial configuration of the simulated box had a size of (5.0×5.0×5.0 nm), with the polymer molecule fixed in the center of the box. 100 TcO_4_^–^ anions, 100 NO_3_^–^ anions, and 3130 H_2_O molecules were prevented around the center, and 200 H_3_O^+^ ions was added to balance the charge of the simulation system. The charmm and GAFF force field were used to describe the whole system. Then in the simulation, the NPT method is used to relax the simulation box. Then, the simulation box was optimized using a canonical integration (NVT) with a time step of 10.0 ps, the temperature was set to 300 K. The optimization time was set to 5000.0 ns, which was enough long for the system to reach a stable system. The trajectory coordinates of the molecules were collected at a storage frequency of 100000 steps. In all atomic dynamics simulations, the motion of the atoms is described by classical Newton's equations and solved using the velocity-Verlet algorithm. All simulations were performed using the gromacs package.


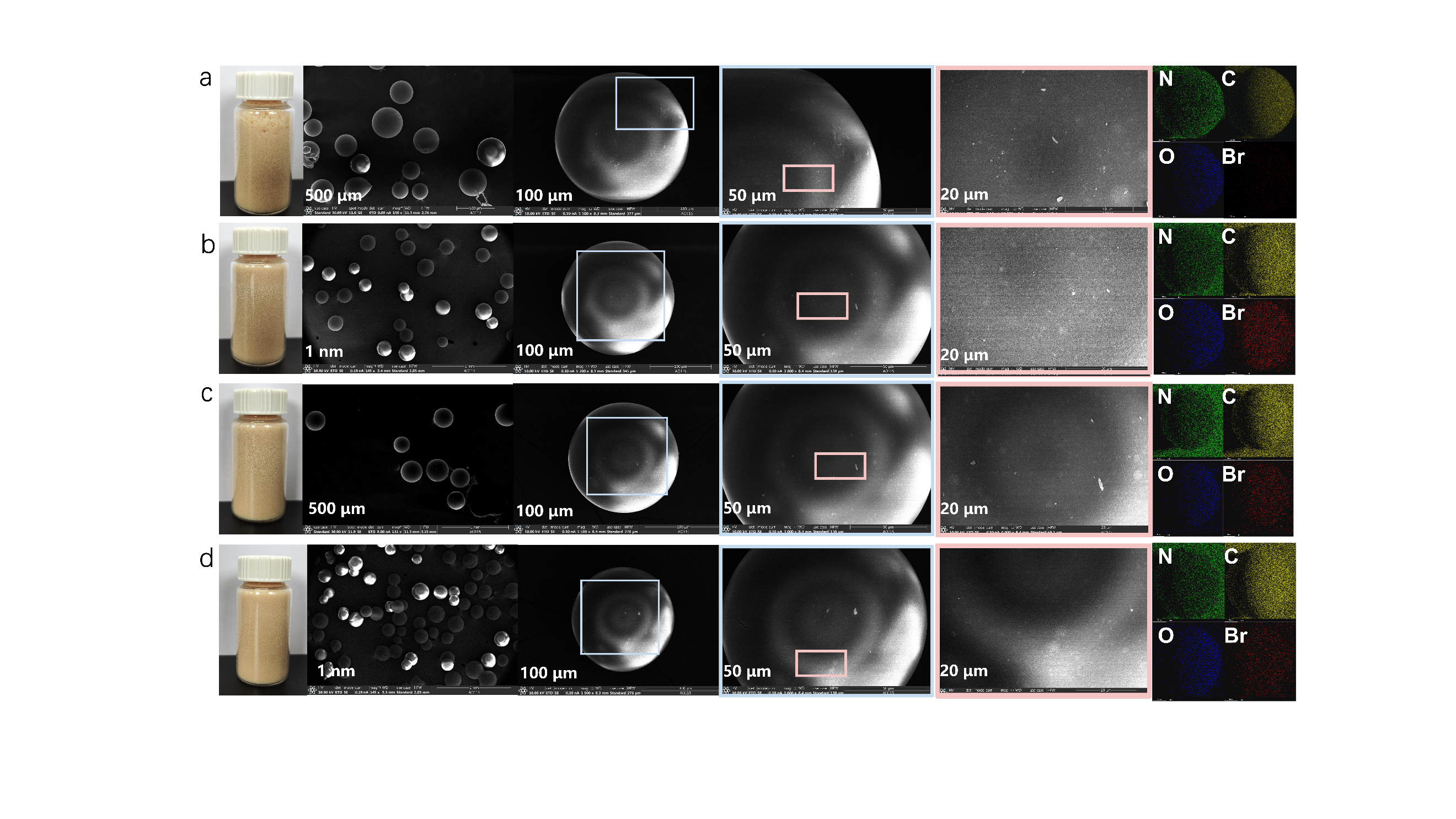


**Figure S1.** Digital photographs, SEM images and EDS spectrogram of C0 PSresin (a), C2 PSresin (b), C5 PSresin (c), C12 PSresin (d). All the scale bars in the insets of SEM images are 1 nm and 500, 100, 50, 20 μm.


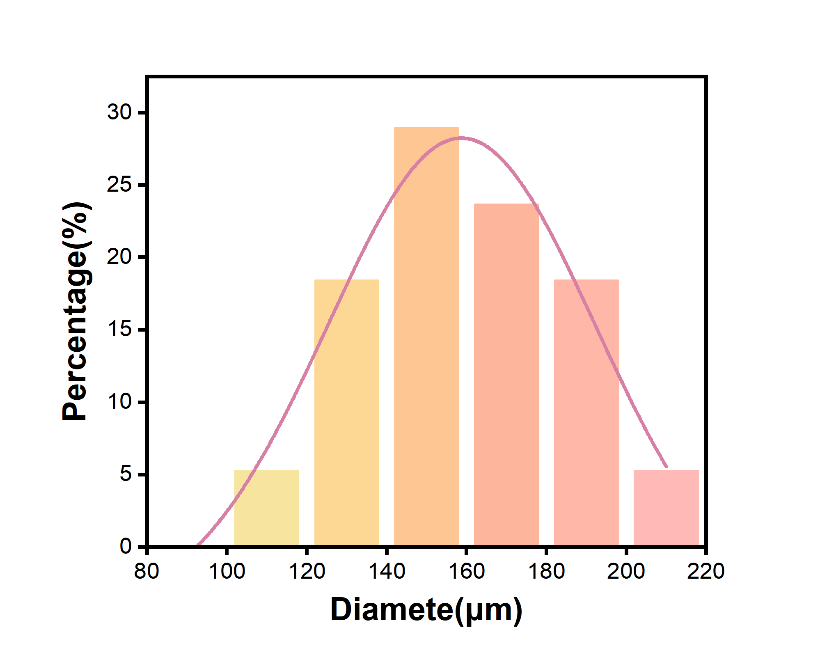


Figure S2. Particle size distribution of vinylpyridine resin.


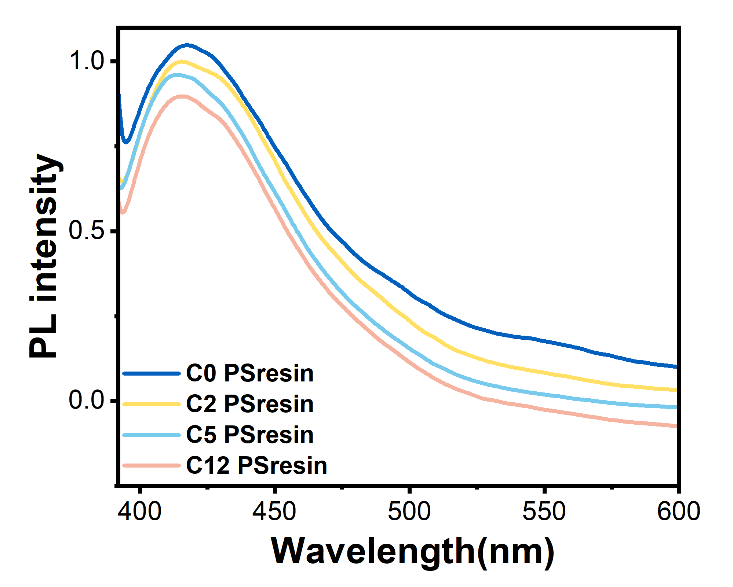


Figure S3. Fluorescence spectra of Cn PSresin.


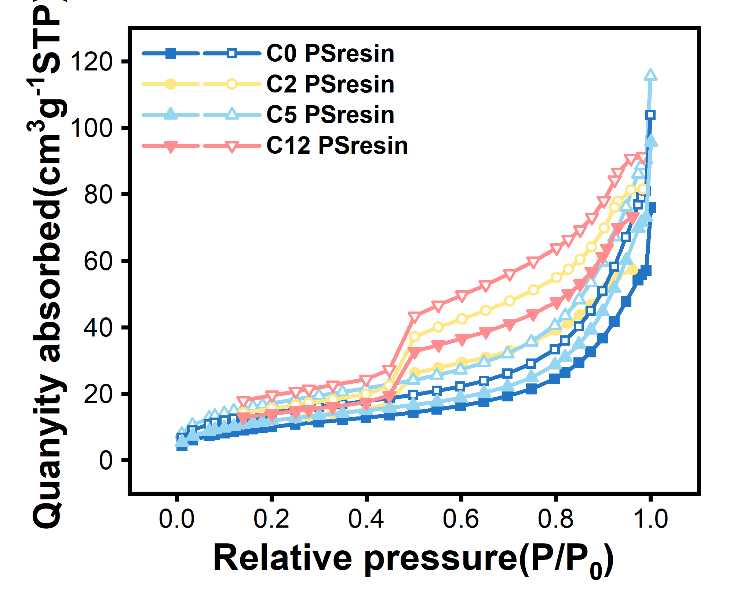


**Figure S4.** N_2_ adsorption–desorption isotherms of Cn PSresin


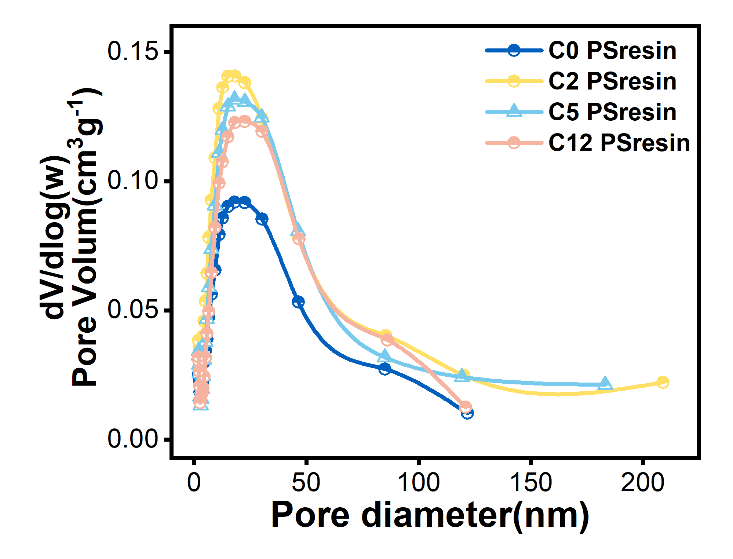


**Figure S5.** The corresponding pore size distributions of Cn PSresin


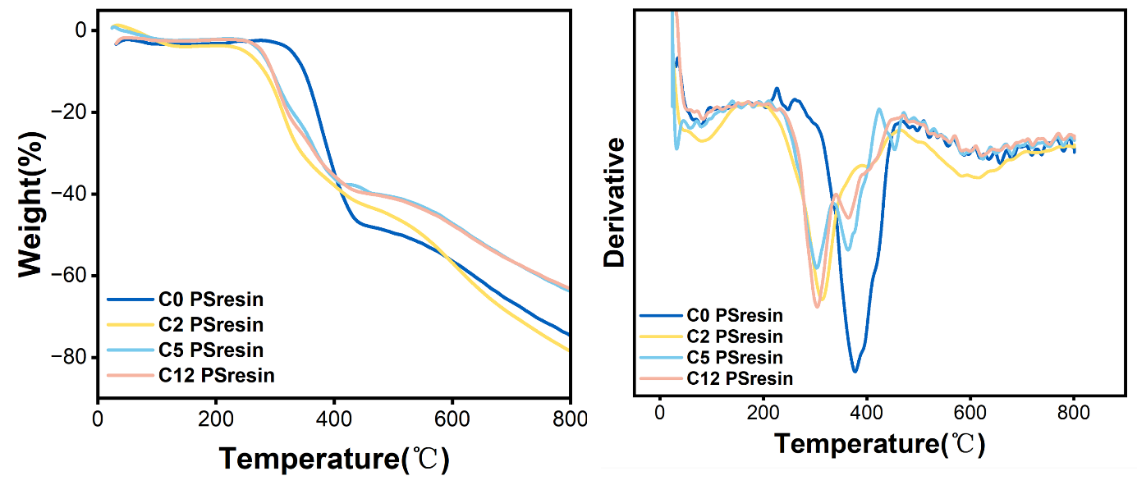


**Figure S6.** The TG and DTG analysis of Cn PSresin.


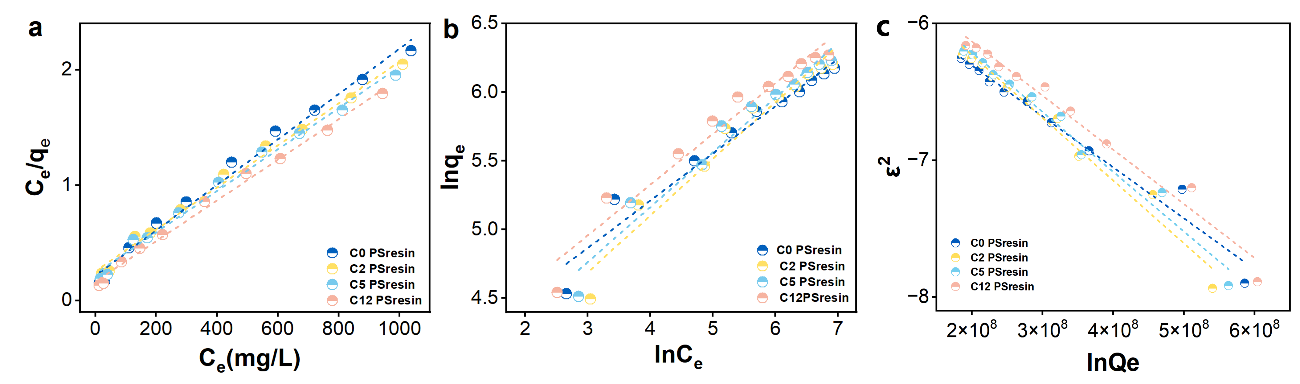


**Figure S7.** The Langmuir fitting curve (a), Freundlich fitting curve (b) and Dubinin-Radushkevich (D-R) model of Cn PSresin at 25℃.

**
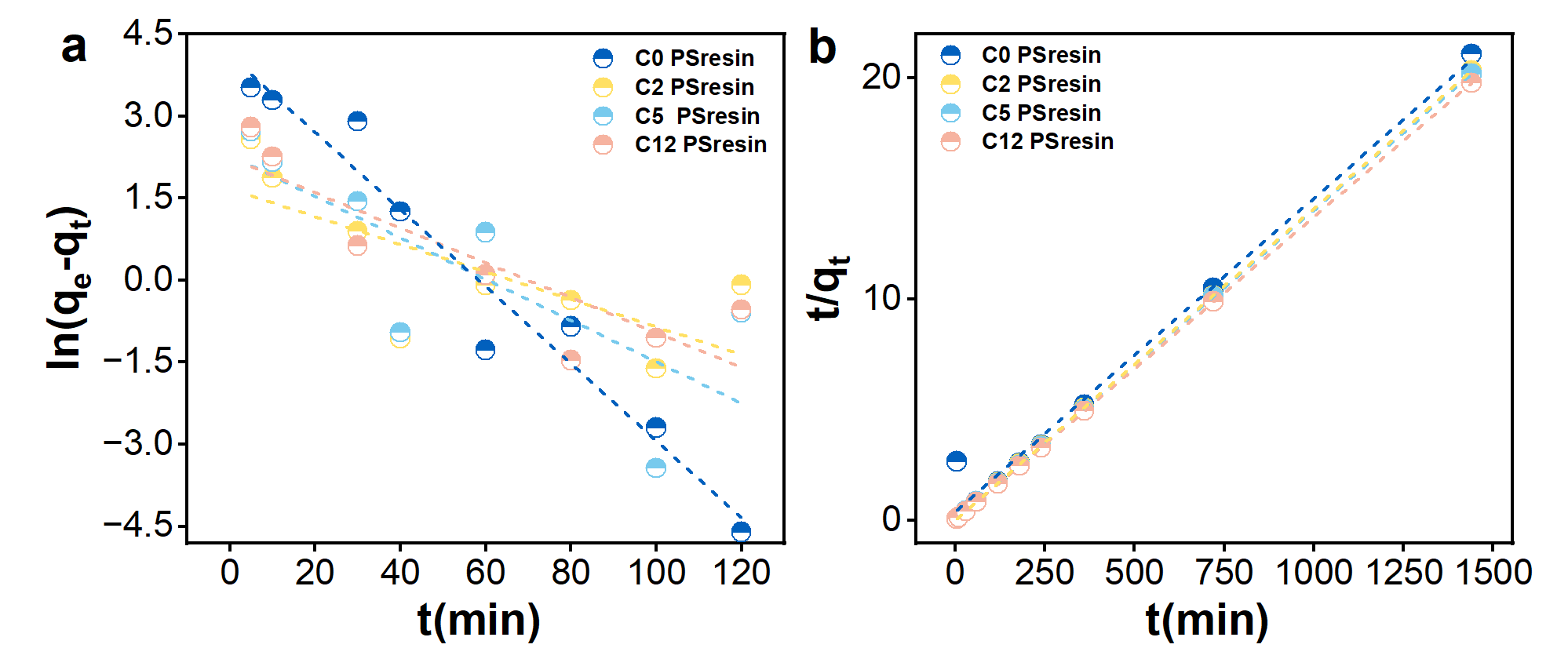
**

**Figure S8.** Pseudo-first/second-order kinetics the corresponding fitting of Cn PSresin.


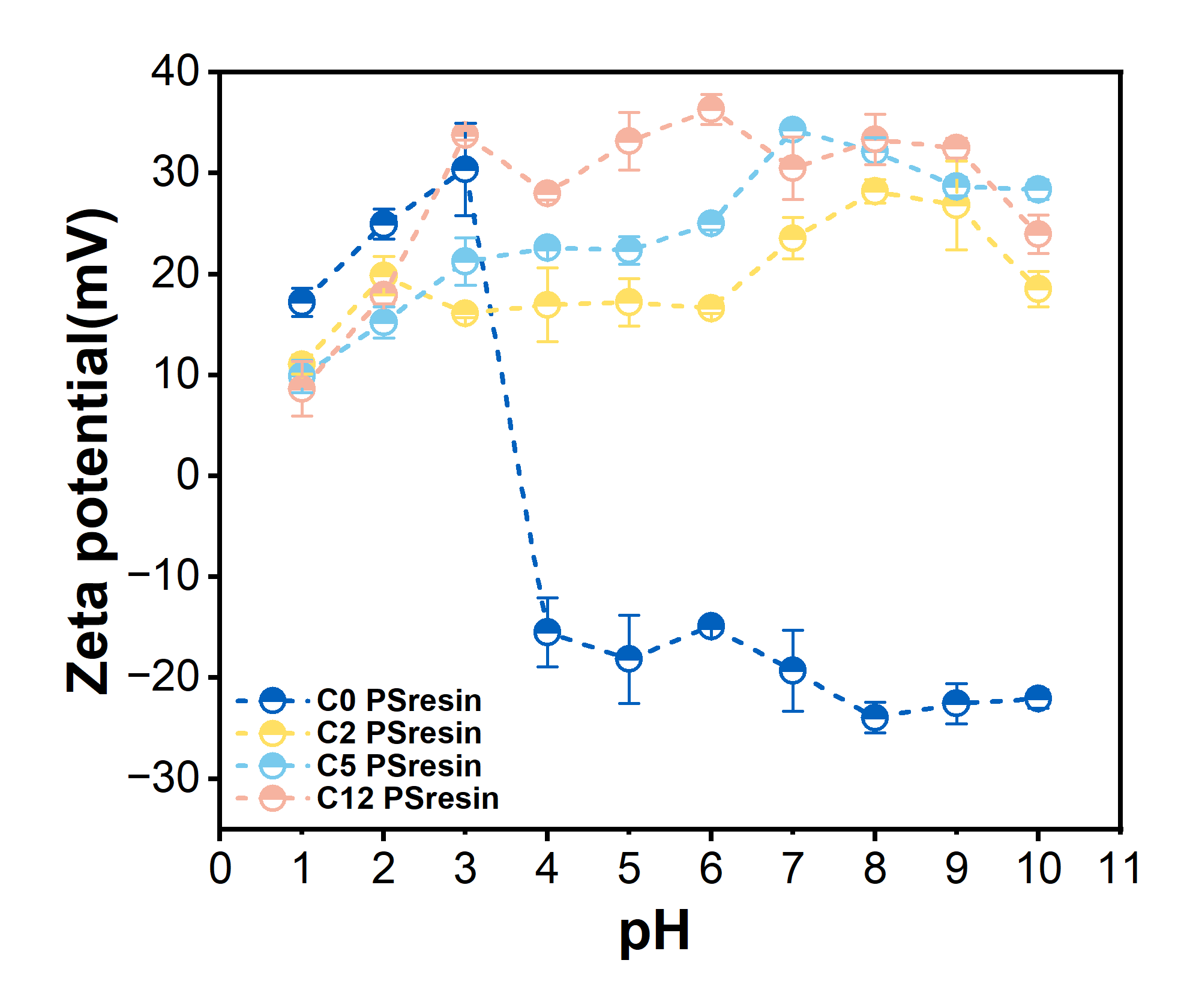


**Figure S9.** Zeta value of PSresin at pH=1-10.

**
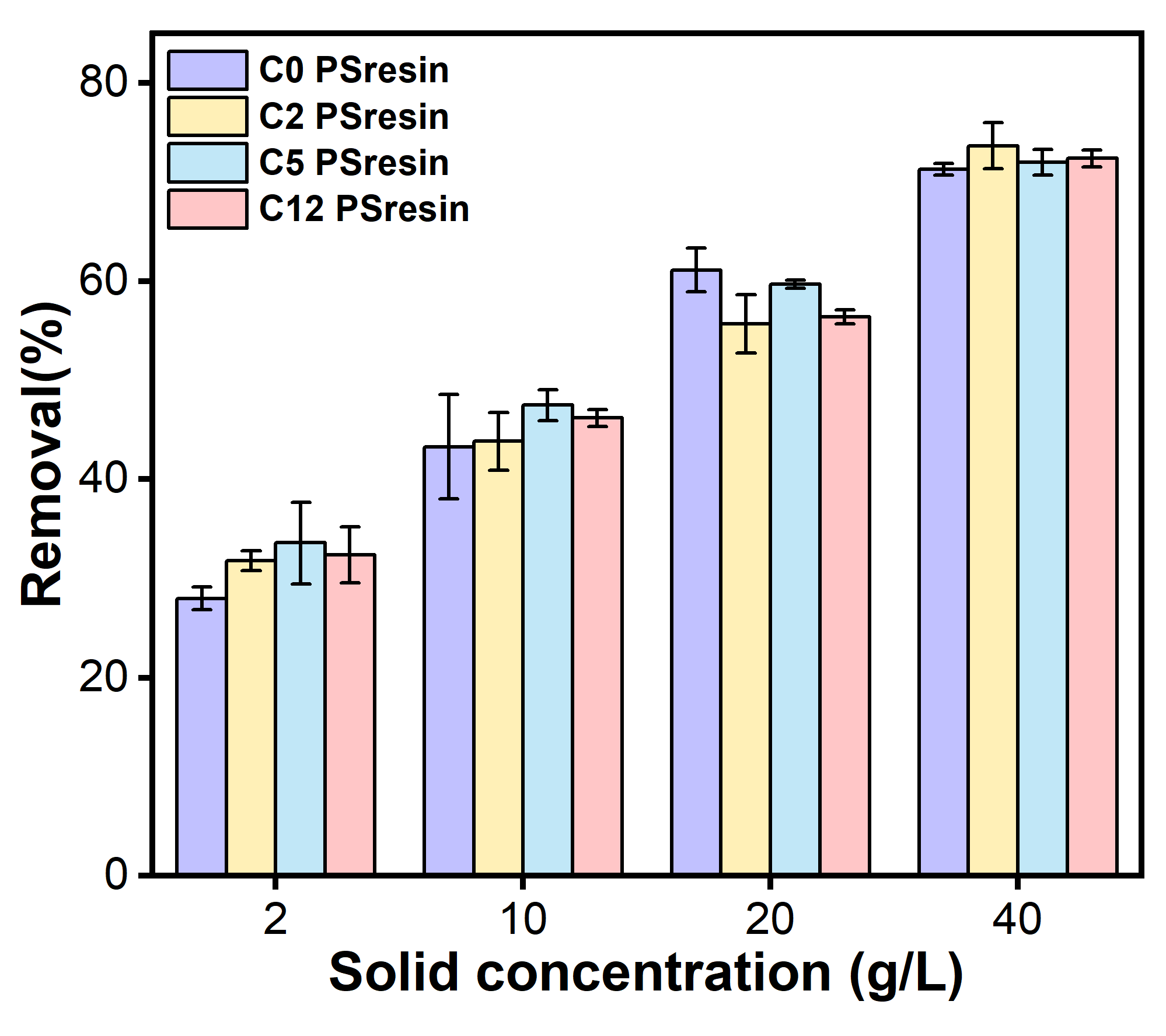
**

**Figure S10.** Effect of different solid-liquid ratios on Cn PSresin adsorption at 3 mol L^-1^ HNO_3_


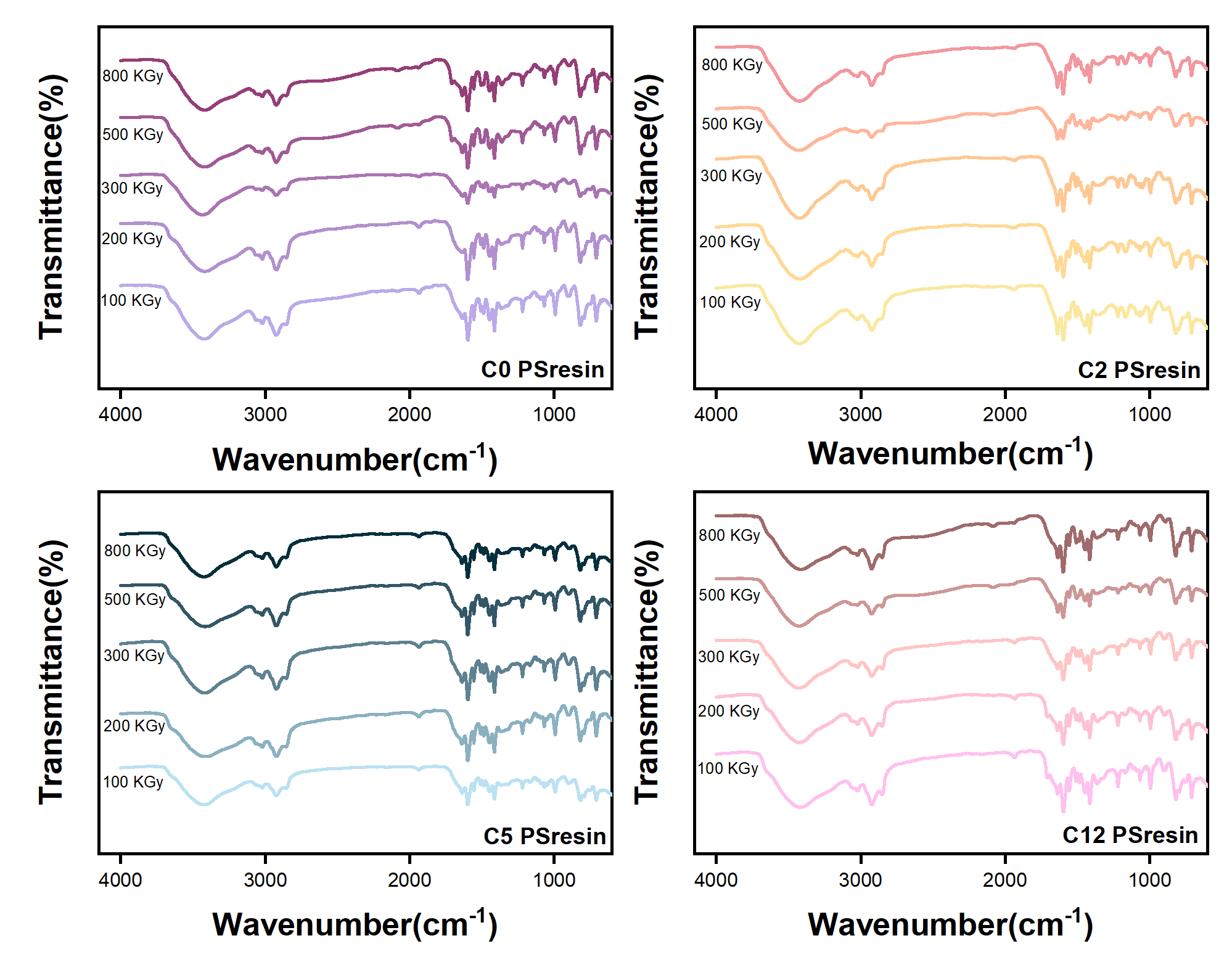


**Figure S11.** FT-IR spectrum of Cn PSresin after β-rays irradiation

**
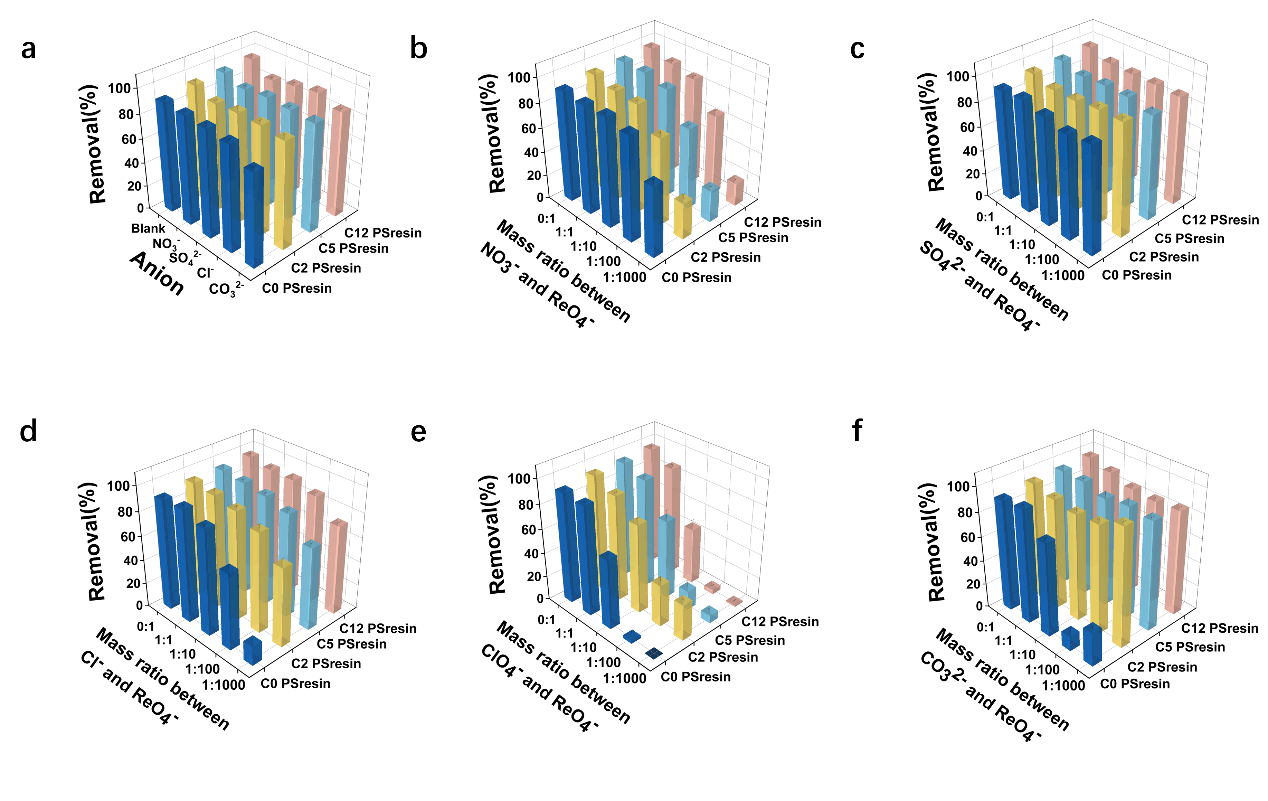
**

**Figure S12.** (a) Effect of competing anions on ReO_4_^–^ removal by Cn PSresin (initially 150 mg L^-1^, mass ratio=1:10) (b), (c), (d), (e), (f) Effect of NO_3_^–^, SO_4_^2–^, Cl^–^, CO_3_^2–^and ClO_4_^–^ on the removal of ReO_4_^–^ (initially 150 pp) by Cn PSresin.


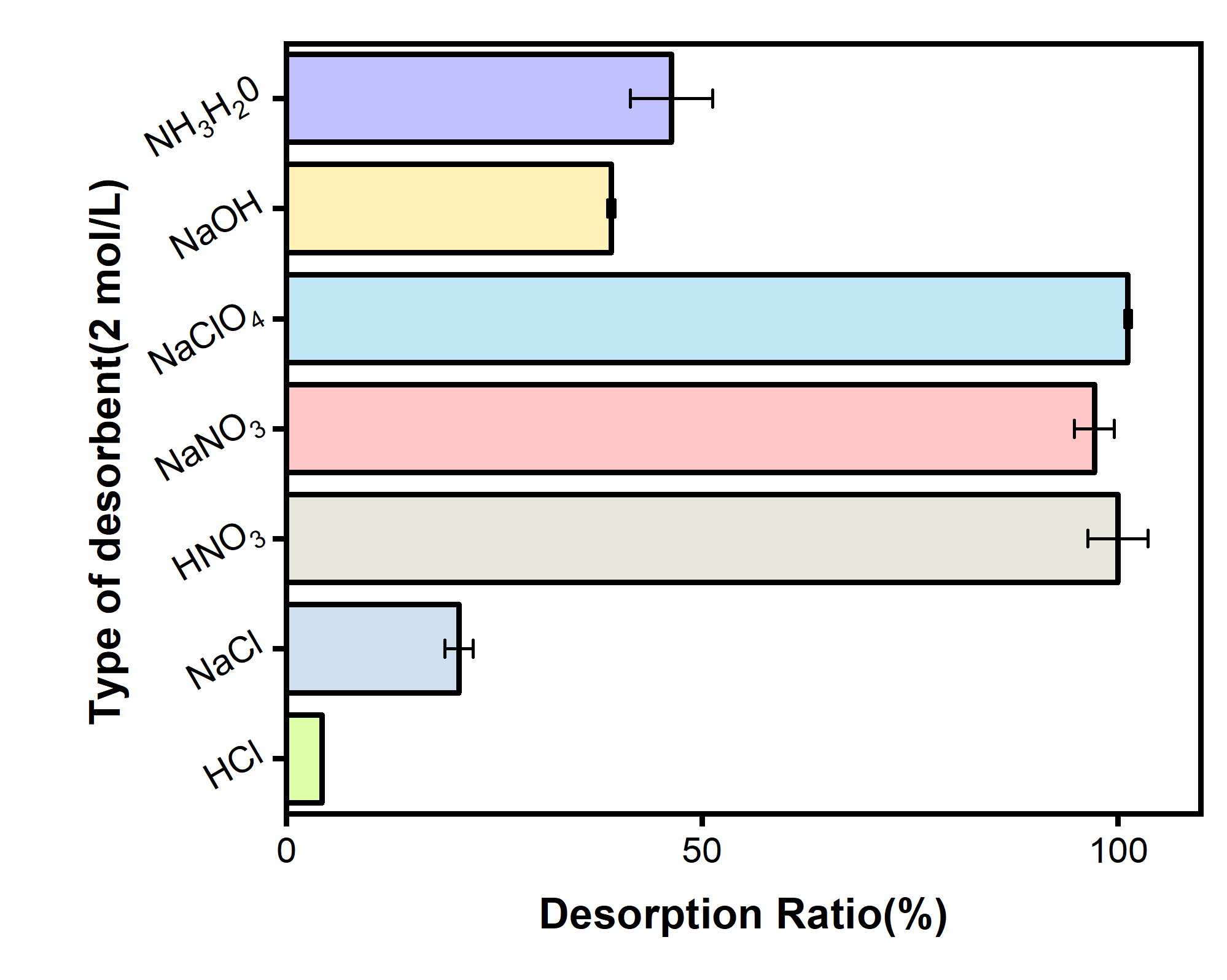


**Figure S13.** Effect of the type of desorption agent on the desorption of PSresin.

**
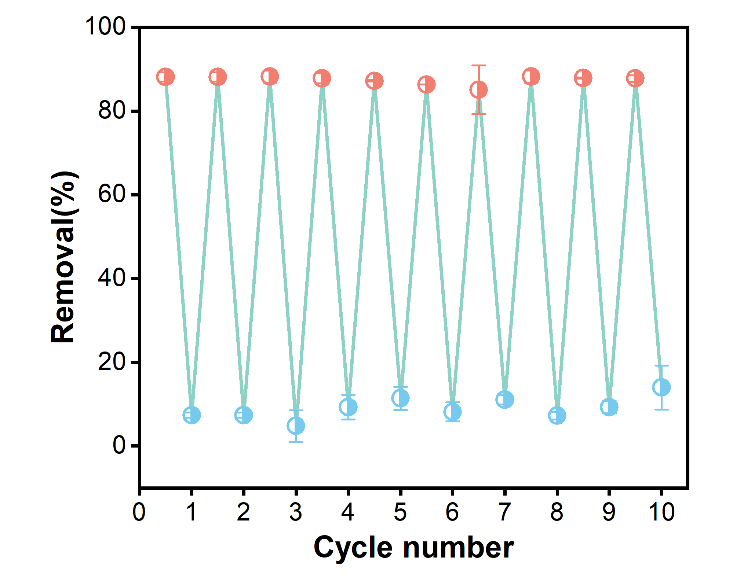
**

**Figure S14.** C2 PSresin reuse performance.

**
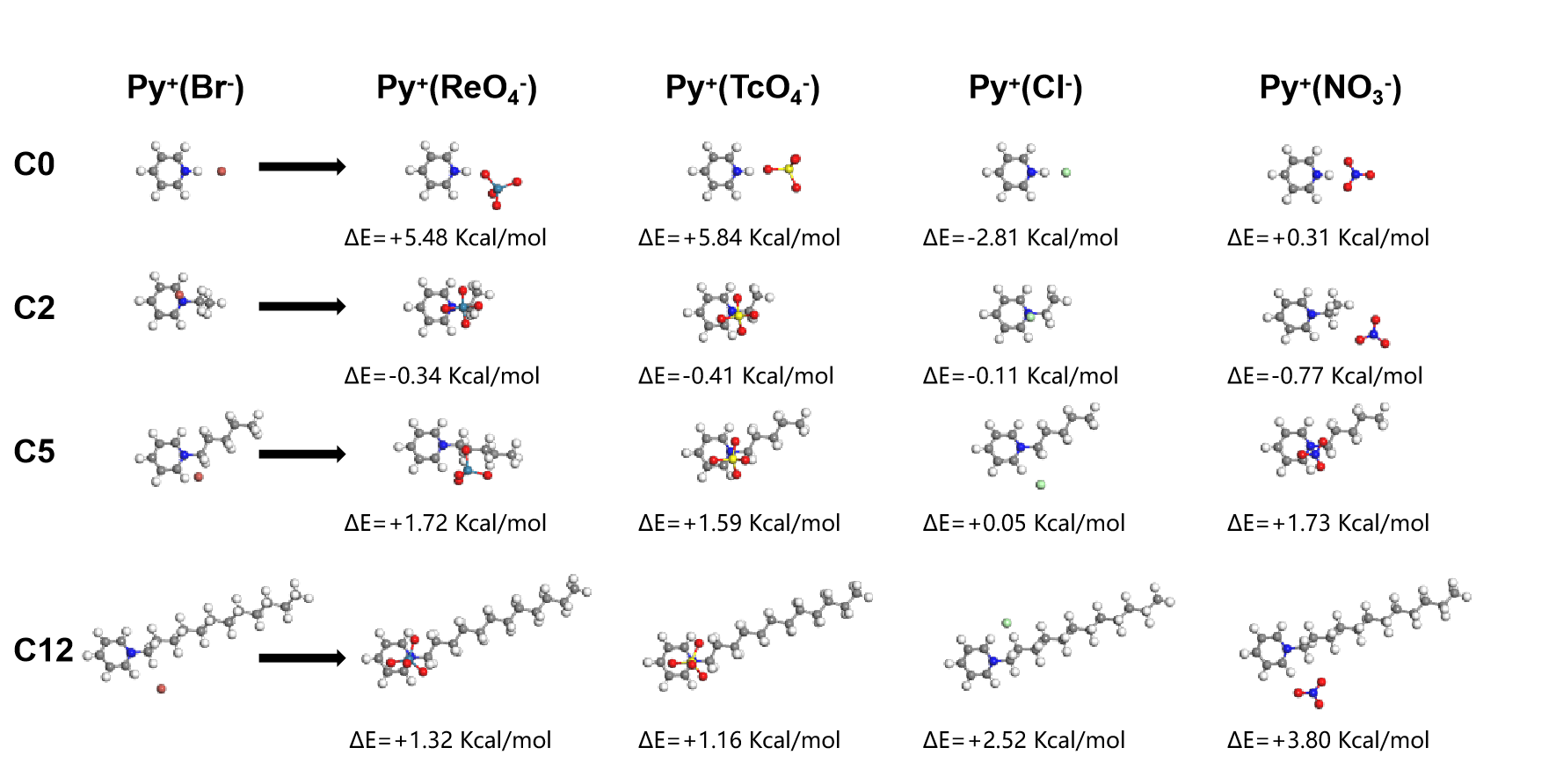
**

**Figure S15.** Results of DFT calculations. The lowest energy configurations and ΔE values of Py_Cn_^+^(Br^–^) and Py_Cn_^+^(A^–^) (A=ReO_4_^–^, TcO_4_^–^, Cl^–^, NO_3_^–^) after optimization.

**
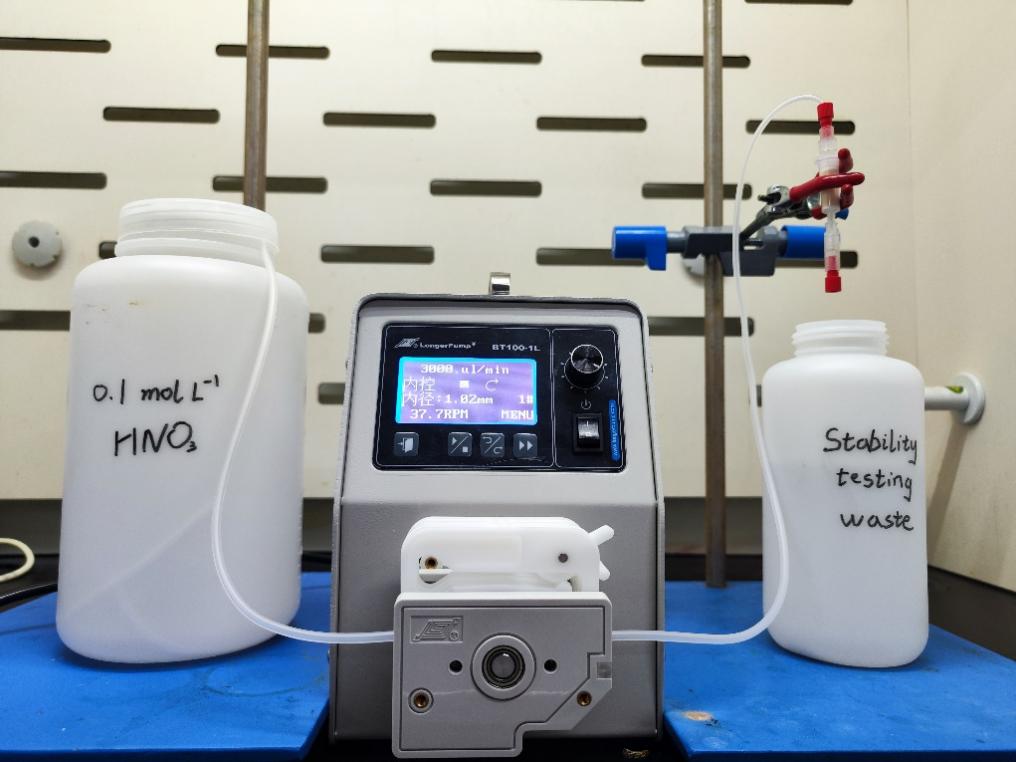
**

**Figure S16** Diagram of the experimental setup for scintillation properties and radiation detection.

**Table S1.** Specific surface areas, pore volumes and modification efficiencies of Cn PSresin.

|  | C0 PSresin | C2 PSresin | C5 PSresin | C12 PSresin |
| --- | --- | --- | --- | --- |
| Specific surface area (m^2^ g^-1^) | 38.07 | 63.85 | 53.35 | 44.72 |
| Pore volume (cm^3^ g^-1^) | 0.11 | 0.17 | 0.16 | 0.16 |
| Quaternary amine nitrogen content | – | 48.76% | 30.86% | 18.80% |

**Table S2.** Comparison of the equilibrium times and adsorption capacities of various adsorbents for ReO_4_^–^.

| Material type | Material | Equilibrium times | capacities  (mg g^-1^) | References |
| --- | --- | --- | --- | --- |
| Resin | C0 PSresin | 40 min | 507.6 | This work |
|  | C2 PSresin | <10 min | 549.5 | This work |
|  | C5 PSresin | <10 min | 558.6 | This work |
|  | C12 PSresin | <10 min | 568.2 | This work |
|  | Purolite A532E | 2.5 h | 446 | [1] |
|  | Purolite A530E | 2.5 h | 706 | [1] |
|  | IRA-401 | 6 h | 330 | [2] |
|  | CMS-g-VBPPh_3_NO_3_-B | 5 h | 251 | [3] |
|  | 4-ATR | 8 h | 354 | [4] |
|  | 4-AMPRs | 15 h | 149.25 | [5] |
|  | PS-g-4VP-IE | 2 h | 252 | [6] |
|  | D318 resin | 6 h | 351 | [7] |
|  | R_2_SO_4_ resin | 4 h | 462 | [8] |
| powdery | PAF-1 | 250 min | 420 | [9] |
|  | SCU-100 | 30 min | 541 | [10] |
|  | SCU-101 | 10 min | 217 | [11] |
|  | SCU-102 | 10 min | 291 | [12] |
|  | SCU-103 | 5 min | 318 | [13] |
|  | SCU-CPN-1 | 10 min | 999 | [14] |
|  | urea-MPN-3 | 30 min | 55 | [15] |
|  | UiO-66-NH_3_^+^Cl^-^ | >24 h | 159 | [16] |
|  | TFPM-PZ-Cl | 30 s | 542.3 | [17] |
|  | NU-1000 | 5 min | 210 | [18] |
| Fiber | PCE fibers | 20 min | 826 | [19] |
|  | NCE fibers | 30 min | 943 | [20] |

**Table S3**. Kinetic parameters for the adsorption of ReO_4_^–^ on Cn PSresin.

|  | PSeudo-first-order | | |  | PSeudo-second-order | | |
| --- | --- | --- | --- | --- | --- | --- | --- |
|  | *q_e_* (mg g^-1^) | *k_1_* (min^-1^) | *R*^2^ |  | *q_e_* (mg g^-1^) | *k_2_* (g mg^-1^ min^-1^) | *R*^2^ |
| C0 PSresin | 61.03 | 0.07046 | 0.9557 |  | 73.80 | 0.0018 | 0.9952 |
| C2 PSresin | 5.283 | 0.02518 | 0.5451 |  | 70.82 | 0.0167 | 0.9999 |
| C5 PSresin | 9.815 | 0.03777 | 0.6061 |  | 72.46 | 0.0092 | 0.9997 |
| C12 PSresin | 9.363 | 0.0320 | 0.7756 |  | 73.26 | 0.0123 | 0.9999 |

**Table S4.** Background count, detection limit and detection efficiency of Cn PSresin.

|  | C0 PSresin | C2 PSresin | C5 PSresin | C12 PSresin |
| --- | --- | --- | --- | --- |
| Background count | 37.7 | 36.12 | 43.26 | 50.3 |
|  | 37.97 | 38.25 | 41.59 | 46.86 |
|  | 38.66 | 38.05 | 43.74 | 48.71 |
|  | 35.96 | 37.11 | 48.71 | 48.32 |
| Detection efficiency (%) | 48.21±2.61 | 44.17±1.86 | 37.50±3.42 | 35.00±2.83 |
| MDA (Bq) | 0.0180 | 0.0196 | 0.0245 | 0.0279 |

**References:**

[1] J. Li, L. Zhu, C. Xiao, L. Chen, Z. Chai, S. Wang, *Radiochim. Acta* **2018**, 106, 581.

[2] H. J. Da, C. X. Yang, X. P. Yan,  *Environ. Sci. Technol.* **2019**, 53, 5212.

[3] Y. Wang, D. H. Han, S. Zhong, X. Li, H. Su, T. Chu, J. Peng, L. Zhao, J. Li, M. Zhai,  *J. Hazard. Mater.* **2020**, 401, 123354.

[4] C. Xiong, C. Yao, X. Wu, *Hydrometallurgy* **2008**, 90, 221.

[5] Z. Dong, J. Liu, D. Wen, M. Zhai, L. Zhao, *J. Hazard. Mater.* **2022**, 433, 128728.

[6] J. Zu, M. Ye, P. Wang, F. Tang, L. He, *RSC Adv.* **2016**, 6, 18868.

[7] Z. Shu, M. Yang, *Chinese J. Chem. Eng.* **2010**, 18, 372.

[8] M. Jia, H. Cui, W. Jin, L. Zhu, Y. Liu, J. Chen, *J. Chem. Technol. Biotechnol.* **2013**, 88, 437.

[9] D. Banerjee, S. K. Elsaidi, B. Aguila, B. Li, D. Kim, M. J. Schweiger, A. A. Kruger, C. J. Doonan, S. Ma, P. K. Thallapally, *Chem. - Eur. J.* **2016**, 22, 17581.

[10] D. Sheng, L. Zhu, C. Xu, C. Xiao, Y. Wang, Y. Wang, L. Chen, J. Diwu, J. Chen, Z. Chai, T. E. Albrecht-Schmitt, S. Wang, *Environ. Sci. Technol.* **2017**, 51, 3471.

[11] L. Zhu, D. Sheng, C. Xu, X. Dai, M. A. Silver, J. Li, P. Li, Y. Wang, Y. Wang, L. Chen, C. Xiao, J. Chen, R. Zhou, C. Zhang, O. K. Farha, Z. Chai, T. E. Albrecht-Schmitt, S. Wang, *J. Am. Chem. Soc.* **2017**, 139, 14873.

[12] D. Sheng, L. Zhu, X. Dai, C. Xu, P. Li, C. I. Pearce, C. Xiao, J. Chen, R. Zhou, T. Duan, O. K. Farha, Z. Chai, S. Wang,  *Angew. Chem., Int. Ed.* **2019**, 58, 4968.

[13] N. Shen, Z. Yang, S. Liu, X. Dai, C. Xiao, K. Taylor-Pashow, D. Li, C. Yang, J. Li, Y. Zhang, M. Zhang, R. Zhou, Z. Chai, S. Wang, *Nat. Commun.* **2020**, 11, 5571.

[14] J. Li, X. Dai, L. Zhu, C. Xu, D. Zhang, M. A. Silver, P. Li, L. Chen, Y. Li, D. Zuo, H. Zhang, C. Xiao, J. Chen, J. Diwu, O. K. Farha, T. E. Albrecht-Schmitt, Z. Chai, S. Wang, *Nat. Commun.* **2018**, 9, 3007.

[15] J. Shen, W. Chai, K. Wang, F. Zhang, *ACS Appl. Mater. Interfaces* **2017**, 9, 22440.

[16] D. Banerjee, W. Xu, Z. Nie, L. E. V. Johnson, C. Coghlan, M. L. Sushko, D. Kim, M. J. Schweiger, A. A. Kruger, C. J. Doonan, P. K. Thallapally, *Inorg. Chem.* **2016**, 55, 8241.

[17] M. Hao, Z. Chen, H. Yang, G. I. N. Waterhouse, S. Ma, X. Wang, *Sci. Bull.* **2022**, 67, 924.

[18] R. J. Drout, K. Otake, A. J. Howarth, T. Islamoglu, L. Zhu, C. Xiao, S. Wang, O. K. Farha, *Chem. Mater.* **2018**, 30, 1277.

[19] R. Zhao, D. Chen, N. Gao, L. Yuan, W. Hu, F. Cui, Y. Tian, W. Shi, S. Ma, G. Zhu, *Adv. Funct. Mater.* **2022**, 32, 2200618.

[20] D. Chen, Z. Liu, S. Li, X. Jing, Y. Tian, W. Hu, F. Cui, R. Zhao, G. Zhu, *Chem. Eng. J.* **2023**, 452, 139148.
